# Machine Learning Potential Analysis of Structural Transition in Cu and Ag Nanoparticles: From Icosahedral to Face-Centered Cubic

**DOI:** 10.1021/acs.jctc.5c00791

**Published:** 2025-08-19

**Authors:** Yongpeng Yang, Jingli Han, Francesc Viñes, Francesc Illas

**Affiliations:** † Henan Institute of Advanced Technology, Zhengzhou University, Zhengzhou 450003, China; ‡ Departament de Ciència de Materials i Química Física & Institut de Química Teòrica i Computacional (IQTCUB), Universitat de Barcelona, c/Martí i Franquès 1-11, 08028 Barcelona, Spain; § School of Material and Chemical Engineering, Zhengzhou University of Light Industry, Zhengzhou 450001, China

## Abstract

A highly accurate high-dimensional neural network potential (HDNNP), trained using more than 180,000 DFT-calculated structures, is used to investigate the structure or realistic Cu–Ag bimetallic particles, as this is the dominant species during the CO_2_ reduction process. The structural transition of Cu and Ag nanoparticles of increasing size, ranging from hundreds of atoms to tens of thousands of atoms, has been studied. Global optimization shows that all Cu and Ag nanoparticles containing 100 to 1000 atoms have an icosahedral core. Upon increasing the number of atoms to 6000 and 10,000 for Cu and Ag, respectively, the nanoparticles’ structural transitions from icosahedral to truncated-octahedral. For even larger nanoparticles, the (100)/(111) surface ratio in truncated-octahedral structures increases, which finally leads to a transformation into the cuboctahedral shape as observed in experiments.

## Graphical abstract

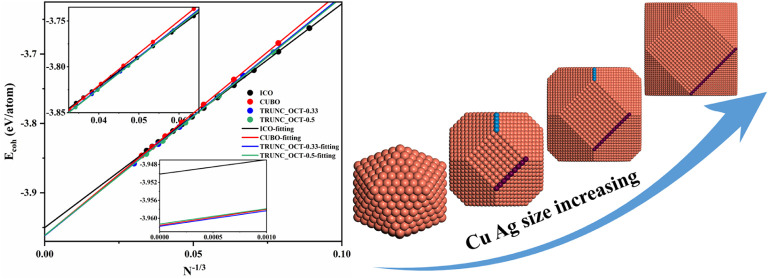

## 1 Introduction

Carbon dioxide (CO_2_) electrocatalytic reduction to various commodity chemicals is a promising way to contribute to decarbonization. Of particular interest is electroreduction to hydrocarbons. However, Cu is so far the only metal which can produce hydrocarbons containing more than two carbons with a high Faradaic efficiency.^1^ However, industrial applications require improving the performance of Cu-based catalysts, in particular, to improve selectivity. In this sense, Cu-based bimetallic catalysts appear as a promising approach to enhance the catalytic performance of these systems by changing the electronic and geometric properties, as well as by generating new active sites.^2–6^ Among various Cu-based bimetallic catalysts, Cu–Ag systems not only exhibit an outstanding catalytic performance for CO_2_ reduction but also suppress the hydrogen evolution reaction (HER), which is an undesired side reaction.^7–10^ Various different Cu–Ag bimetals have been synthesized and applied to CO_2_ electroreduction, including alloys, intermetallic compounds, and phase-separated systems.^11–14^ Because the formation of Cu–Ag alloys is thermodynamically unfavorable, only a small amount of Cu–Ag bimetallic systems is usually obtained. In spite of this limitation, the Cu–Ag nanorods synthesized by Yu et al.^15^ have two times higher selectivity toward C_2_ products than pure Cu at −1.0 V vs RHE. Also remarkable is the work of Li et al.,^16^ who prepared bulk-miscible Cu–Ag alloys by the cosputtering approach, and Ag_0.14_Cu_0.86_ achieved a record Faradaic efficiency of 41% toward ethanol at 250 mA/cm^2^ and −0.67 V vs the reversible hydrogen electrode (RHE). Cu@Ag core–shell nanocatalysts capped with oleylamine exhibit high CO_2_ reduction activity and selectivity toward ethylene.^17^ Notably, tandem Cu–Ag nanocatalysts also have four times higher selectivity for C_2_ compounds compared to individual Cu nanocatalysts.^18^

The catalytic performance of Cu–Ag bimetals depends on many factors such as the Cu/Ag ratio, morphology, and size. Moreover, the reconstruction of bimetallic nanoparticles under reaction conditions is very common, and the redistribution of metal atoms and structural transformations are mainly driven by adsorbates such as H*, O*, and CO*.^19,20^ In heterogeneous thermocatalysis, the reconstruction of bimetallic nanoparticles usually occurs at rather high temperatures; it can also take place at moderate temperatures^21^ or as a result of synthetic conditions.^22^ For example, Chen et al.^23^ observed a high mobility of Cu to the surface in Cu–Ag bimetallic nanoparticles triggered by CO_2_ reduction. Thus, a precise control of bimetallic nanoparticles is very challenging while it is required to optimize their stability and catalytic performance.^24^ There is also compelling evidence that CO can cause the segregation for many bimetallic systems such as Pd–Au or Pd–Ag.^25–27^ As an important adsorbate and product of CO_2_ reduction, CO also plays a critical role in the segregation of Cu–Ag. However, the details of the segregation process of the Cu–Ag bimetal driven by CO adsorption remain largely unknown.

Recently, the development of machine learning potentials provides a promising opportunity to investigate highly complex processes by integrating molecular dynamics, Monte Carlo simulations, and other computational approaches. These new approaches make it possible to carry out long-enough time-scale simulations for large systems with accuracy comparable to that provided by the density functional theory (DFT)-based calculations used in the potential training. In this work, we trained a high-dimensional neural network potential (HDNNP) for the Cu–Ag–CO system using a large amount of DFT-calculated data using the Perdew–Burke–Ernzerhof (PBE) exchange–correlation functional.^28^ Next, the high accuracy of the obtained HDNNP was confirmed by comparing predictions to DFT calculations for systems not included in the training set. Using this HDNNP, it is possible to study realistic systems with a size similar to that of the synthesized metal nanoparticles containing several thousands of atoms, thus going much beyond previous studies of Cu and Ag nanoparticles involving a few hundred atoms only.^29–32^ By means of the obtained HDNNP, we studied the structural transition of Cu and Ag nanoparticles with increasing size, ranging from hundreds of atoms to tens of thousands of atoms, finding that all Cu and Ag nanoparticles containing 100 to 1000 atoms have an icosahedral core; that nanoparticles with around 6,000 and 10,000 atoms for Cu and Ag, respectively, become truncated-octahedral; and that for even larger nanoparticles, the cuboctahedral shape is preferred in agreement with experiments.^33–37^

## 2 Computational Details

### 2.1 Density Functional Calculations

All DFT-based calculations were performed using the CP2K code.^38^ To carry out the calculation, the Perdew–Burke–Ernzerhof (PBE) exchange–correlation functional^28^ and double-ζ valence polarized (DZVP) molecularly optimized basis sets combining with Geodcker–Teter–Hutter (GTH) pseudopotentials were chosen.^39,40^ PBE is known for being one of the best functional in describing transition metal (TM) bulks and surfaces,^41,42^ and so likely, TM nanoparticles.^43^ The plane-wave cutoff energy was set at 500 Ry, and the self-consistent field (SCF) convergence was set at 1.0 × 10^–6^ Ha. Since the systems studied in the present work are all finite, the calculations were carried out at the γ point. Although the main aim of the present work is to describe the relative stability of Cu, Ag, and bimetallic Cu–Ag nanoparticles, calculations also involved the structure covered by CO so as to generate the high-dimensional neural network potential that, in a subsequent step, can be applied to study the influence of CO adsorption on their structural stability. For C, O, Cu, and Ag, 4, 6, 11, and 11 valence electrons were considered, respectively. The D3 method with Becke–Johnson damping, hereafter denoted as PBE-D3BJ, was employed to account for the van der Waals interaction.^44^

### 2.2 High-Dimensional Neural Network Potential Setup and Training

The training of the machine learning potential was performed using the n2p2 code.^45^ The high-dimensional neural network potential contains two hidden layers, each containing 20 nodes. We used the radial symmetry function (*G*_*i*_^2^) and angular symmetry function (*G*_*i*_^3^) presented by Behler and Parrinello^46^ as

1 $$G_{i}^{2}=\sum_{j\neq i}e^{-\eta {\left(r_{\mathrm{ij}}-r_{s}\right)}^{2}}f_{c}\left(r_{\mathrm{ij}}\right)$$

2 $$G_{i}^{3}=2^{1-\zeta }\sum_{\begin{array}{l} j,k\neq i\\ j<k \end{array}}{\left(1+\lambda \cos\theta_{\mathrm{ijk}}\right)}^{\zeta }e^{-\eta \left({\left(r_{\mathrm{ij}}-r_{s}\right)}^{2}+{\left(r_{\mathrm{ik}}-r_{s}\right)}^{2}+{\left(r_{\mathrm{jk}}-r_{s}\right)}^{2}\right)}f_{c}\left(r_{\mathrm{ij}}\right)f_{c}\left(r_{\mathrm{ik}}\right)f_{c}\left(r_{\mathrm{jk}}\right)$$

where *f*_*c*_ is a cutoff function given as

3 $$f_{c}\left(r\right)=\tanh^{3}\left(1-\frac{r}{r_{c}}\right)$$

In these equations, *r*_*ij*_ represents the distance between atoms *i* and *j*, and θ_*ijk*_ is the angle composed by the atoms labeled *i*, *j*, and *k*. For the angular symmetry function, the *r*_*s*_ value in this work was taken as zero, with λ being equal to 1 or −1. All of the η and ζ values were automatically generated by using the strategy presented by Imbalzano et al.,^47^ and the cutoff radius was 14 Bohr. More than 1000 symmetry functions were generated by setting the number of parameter pairs to 20, and 600 symmetry functions were finally selected, which contains 120 radial symmetry functions, 82 shifted symmetry functions, and 398 narrow angular symmetry functions. In addition, 150, 149, 152, and 149 symmetry functions were used for C, O, Cu, and Ag elements, respectively.

The committee methodology presented by Schran et al.^48^ was used to improve the accuracy of the HDNNP. The committee disagreement provides a way to monitor and control the accuracy of the model relative to its parent ab initio method and its training set. Besides, the computational cost is almost unaffected when committee-HDNNP calculations are performed compared with a single HDNNP, except for training the HDNNP multiple times at the beginning. We trained eight HDNNPs by changing the random number generator seed with the same training data for the committee-HDNNP.

The training data was generated employing the minima hopping method. This approach can efficiently sample different structures by initializing the velocities along low-curvature directions to cross rapidly over small energy barriers into neighboring basins, and temperature information was included ranging from zero to above the melting point. The minima hopping method was combined with NVE-ensemble molecular dynamics simulations of 150 to 200 steps and geometry optimization for each iteration, with initial MD temperatures set to 1000–2000 K for efficient sampling, and the number of softening iterations was set to 10 to initialize the velocities. The initial nanoparticle models were based on icosahedral structures, while the initial bulk and slab models adopted FCC-type configurations. All newly obtained structures were accepted for subsequent searches during minima hopping iterations, regardless of whether their energies increased or decreased. About 1000 to 2000 structures were generated for each minima hopping simulation. A total of 185,739 structures were considered, including 7,025,0642 atomic configurations. The icosahedral metal nanoparticles with 55, 147, 309, and 561 atoms were selected as initial structures with one additional metal adatom for the spin-unpolarized calculation. After minima hopping simulations, all of the geometric structures of the icosahedral metal nanoparticles change significantly (see Figure S1 of the Supporting Information, SI). Pure Cu, pure Ag, and Cu–Ag bimetallic systems were all considered, and there were 77,298 structures including information from 20,465,208 atomic configurations. For the Cu–Ag bimetallic nanoparticles, as shown in Figure 1, Cu@Ag and Ag@Cu core–shell structures, Cu/Ag segregated structures, and Cu–Ag random alloy structures with various shell thicknesses and Cu/Ag ratios were considered. Various bulk and periodic slab models, including (100) and (111) surfaces, were also selected as initial structures for minima hopping simulations. It is noted that (100) and (111) surfaces get rough after several minima hopping simulation steps (*cf*. Figure S2), suggesting the sampling efficiency of the minima hopping method.

**Figure 1:**
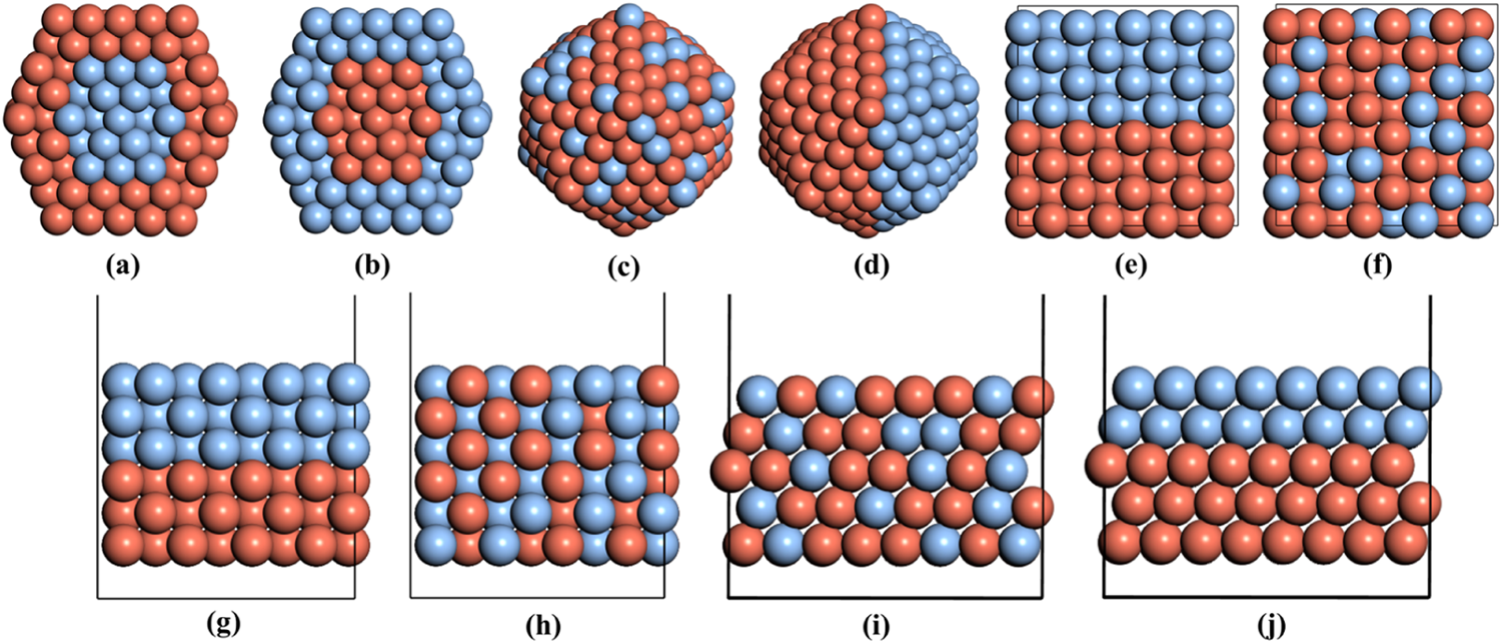
Snapshots of Cu–Ag bimetal models as initial structures for training data generation: Cu@Ag core–shell (a), Ag@Cu anti-core–shell (b), random alloy (c), and segregated nanoparticle (d) structures; Cu–Ag segregated (e) and random alloy (f) bulk structures; Cu–Ag segregated (g) and random alloy (h) (100) surfaces; and Cu–Ag segregated (i) and random alloy (j) (111) surfaces. Cu and Ag atoms are shown as orange or blue spheres, respectively.

To derive the high-dimensional neural network potential that will be able to describe not only the naked particles but also, in a subsequent work, those covered with different CO coverages, Cu–CO, Ag–CO, and Cu–Ag–CO systems were considered. To study CO adsorption, the selected metal nanoparticle models were the same as those previously considered without CO. The CO adsorption coverage situation considered ranges from 0.1 to 1.0, and a total of 108,441 structures including 49,785,434 atomic information were taken into account. During the HDNNP training, 90% of the data were selected as training data, and the remaining 10% of the data were testing data.

## 3 Results and Discussion

### 3.1 Comparison of Computational Results of the HDNNP and DFT

The root-mean-square error (RMSE) of energy and forces is summarized in Table S1. For the eight HDNNPs, the RMSE of energy for the training set ranges from 1.48 to 1.60 meV/atom, and the RMSE of force ranges from 83.7 to 92.7 meV/Å, both of which are very close to those of the testing set. The small RMSEs of energy and forces indicate that the HDNNPs have high accuracy, and they are not overfit (*cf*. Figure 2).

**Figure 2:**
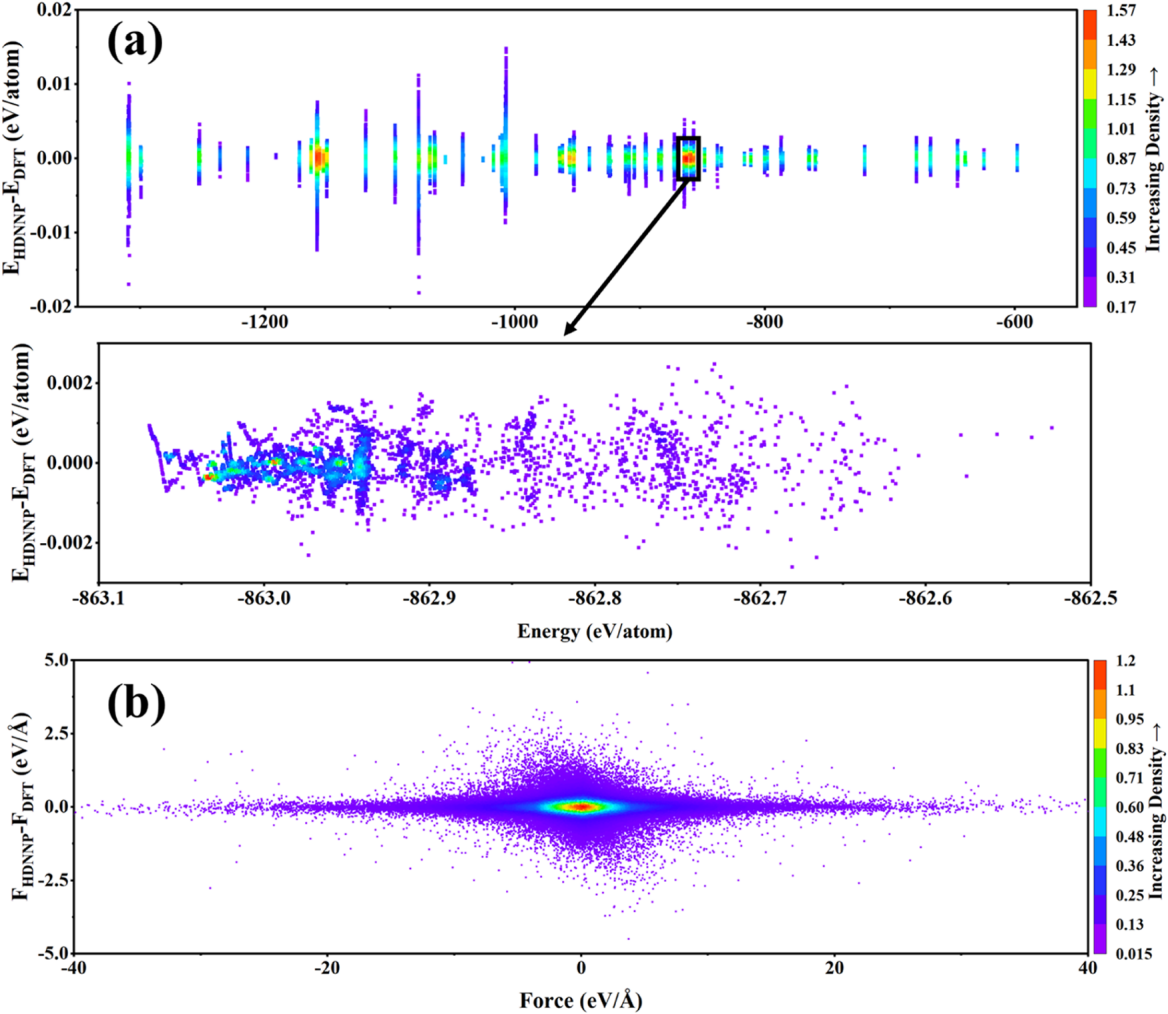
HDNNP error in total energy (a) and forces (b) relative to DFT results.

To further validate the accuracy of HDNNPs, we compared the cell parameters and average cohesive energies of bulk Ag and Cu as calculated by HDNNPs and DFT. Additionally, we examined the influence of dispersion forces on these properties. As summarized in Table 1, the Cu cell length at 0 K predicted by HDNNPs is 3.567 Å, closely aligning with the PBE-D3BJ result of 3.597 Å. Under NPT ensemble molecular dynamics at 300 K using HDNNPs, this value slightly increases to 3.592 Å, only 0.021 Å smaller than the experimental measurement of 3.613 Å at 293 K. The cohesive energy calculated by HDNNPs is −3.96 eV, almost identical to the PBE-D3BJ result, as expected, but 0.47 eV higher than the experimental value of −3.49 eV. Without considering dispersion forces, the cohesive energy is −3.44 eV, which is much closer to the experimental value. However, the length of the Cu cell at 0 K is 3.651 Å, and the difference compared with experimental results can be much higher than 0.038 Å after considering the thermal expansion. These results suggest that while the PBE functional combined with DFT-D3BJ parameters offers accurate geometrical predictions for Cu, it tends to overestimate the cohesive energy.

**Table 1:** Comparison of Lattice Parameters (*a*_0_ and *a*_300_ in Å, at 0 and 300 K, Respectively) and Cohesive Energy (*E*_coh_ in eV/atom) for Bulk Cu and Ag Calculated Using PBE-D3BJ, HDNNP, PBE, and Experimental Results, Where *a*_293_ Is the Lattice Constant at 293 K

	PBE-D3BJ		HDNNP			PBE		experiment	
	*a*_0_	*E*_coh_	*a*_0_	*a*_300_	*E*_coh_	*a*_0_	*E*_coh_	*a*_293_	*E*_coh_
Cu	3.597	3.962	3.567	3.592	–3.962	3.651	–3.44	3.613	–3.49
Ag	4.101	–3.008	4.088	4.113	–3.006	4.156	–2.51	4.086	–2.95

For bulk Ag, HDNNPs predict a cell length of 4.088 Å at 0 K, which closely matches the PBE-D3BJ result of 4.101 Å. At 300 K, the NPT ensemble molecular dynamics simulation with HDNNPs gives a slightly increased cell length of 4.113 Å, just 0.027 Å larger than the experimental value of 4.086 Å at 293 K. The cohesive energy predicted by HDNNPs is −3.01 eV, nearly identical to the PBE-D3BJ result and only 0.06 eV higher than the experimental value of −2.95 eV. Notably, when dispersion forces are excluded, the cohesive energy drops to −2.51 eV, which is 0.44 eV lower than the experimental value, indicating the need to account for dispersion. Furthermore, the length of the Ag cell at 0 K is 4.156 Å, and the difference compared to experimental results can be as high as 0.07 Å after considering the thermal expansion. Thus, the PBE-D3BJ functional yields accurate predictions for both the geometric structure and the cohesive energy of bulk Ag.

We also tested the accuracy of the HDNNP for Ag and Cu nanoparticles. Specifically, the cohesive and relative energies of icosahedral and cuboctahedral nanoparticles with 55, 309, and 923 atoms were calculated, with the results summarized in Tables 2 and 3. Regarding the cohesive energy, the HDNNP value for icosahedral Cu_55_ (labeled as Cu_55_-ICO) is −3.091 eV/atom, which is only 0.009 eV/atom higher than that obtained by PBE-D3BJ. For cuboctahedral Cu_55_ (labeled as Cu_55_-CUBO), the cohesive energy difference is 0.014 eV/atom. The PBE-D3BJ calculations predict that the energy of Cu_55_-ICO is 3.78 eV lower than Cu_55_-CUBO, while the HDNNP value is 3.44 eV. For Cu_309_-ICO, the HDNNP cohesive energy is only 0.003 eV/atom higher than the PBE-D3BJ value, while it is 0.010 eV/atom higher for Cu_309_-CUBO. In a similar way, the PBE-D3BJ total energy of Cu_309_-ICO is 11.80 eV lower than that of Cu_309_-CUBO, with the HDNNP value being 9.71 eV. Interestingly, the HDNNP cohesive energy of Cu_923_-ICO is lower by 0.004 eV/atom than the PBE-D3BJ result, and it is lower by only 0.001 eV/atom for Cu_923_-CUBO. The total energy of Cu_923_-ICO is lower by 21.19 and 17.75 eV than Cu_923_-CUBO, according to DFT and the HDNNP, respectively. It is worth pointing out that these cuboctahedral structures were not included in the training set used to develop the HDNNP, and the HDNNP still calculated these new structures very well. Furthermore, when the HDNNP structures are recalculated with PBE-D3BJ in a single-point fashion, the obtained energies are very close to those of the optimized geometry, and the energy difference is within 0.002 eV/atom, not only for the icosahedral nanoparticles but also for the cuboctahedral nanoparticles.

**Table 2:** Comparison of PBE-D3BJ-, HDNNP-, and PBE-Calculated Results of Icosahedral (ICO) and Cuboctahedral (CUBO) Cu Nanoparticles^a^

	*E*_coh_				Δ*E*		
	PBE-D3BJ	PBE-D3BJ^NN^	NNP	PBE	PBE-D3BJ	NNP	PBE
Cu_55_-ICO	–3.082	–3.081	–3.091	–2.819	0	0	0
Cu_55_-CUBO	–3.013	–3.011	–3.028	–2.756	3.78	3.44	3.29
Cu_309_-ICO	–3.468	–3.467	–3.471	–3.099	0	0	0
Cu_309_-CUBO	–3.430	–3.428	–3.440	–3.067	11.80	9.71	9.71
Cu_923_-ICO	–3.623	–3.622	–3.618	–3.208	0	0	0
Cu_923_-CUBO	–3.600	–3.598	–3.599	–3.190	21.19	17.75	16.96

a The PBE-D3BJ^NN^ column reports the value calculated at the HDNNP-predicted geometry. *E*_coh_ values are in eV/atom and Δ*E* is the energy difference with respect to the most stable isomer in eV.

**Table 3:** Comparison of PBE-D3BJ-, HDNNP-, and PBE-Calculated Results of Icosahedral (ICO) and Cuboctahedral (CUBO) Ag Nanoparticles^a^

	*E*_coh_				Δ*E*		
	PBE-D3BJ	PBE-D3BJ^NN^	NNP	PBE	PBE-D3BJ	NNP	PBE
Ag_55_-ICO	–2.331	–2.330	–2.338	–2.054	0	0	0
Ag_55_-CUBO	–2.284	–2.283	–2.293	–2.014	2.56	2.46	2.23
Ag_309_-ICO	–2.629	–2.629	–2.632	–2.258	0	0	0
Ag_309_-CUBO	–2.604	–2.604	–2.611	–2.238	7.75	6.57	5.82
Ag_923_-ICO	–2.753	–2.752	–2.746	–2.342	0	0	0
Ag_923_-CUBO	–2.736	–2.735	–2.733	–2.330	15.80	12.69	11.26

a The PBE-D3BJ^NN^ column reports the value calculated at the HDNNP-predicted geometry. *E*_coh_ values are in eV/atom and Δ*E* is the energy difference with respect to the most stable isomer in eV.

The HDNNP cohesive energy of icosahedral Ag_55_-ICO is −2.338 eV/atom, which is only 0.007 eV/atom higher than the PBE-D3BJ value. A similar result is found for the cuboctahedral Ag_55_-CUBO particle, where the difference in cohesive energy is 0.009 eV/atom with HDNNP and PBE-D3BJ cohesive energy values of 2.46 and 2.56 eV, respectively. The same degree of accuracy is found for Ag_309_-ICO, the cohesive energy difference being 0.002 eV/atom only with the HDNNP value being slightly higher. For the larger Ag_309_-CUBO nanoparticles, the difference in cohesive energy is still quite small (0.007 eV/atom) with the HDNNP value being again larger. For the Ag_309_-ICO particle, the cohesive energy is lower than that of Ag_309_-CUBO by 7.75 eV and is 6.57 eV at the PBE-D3BJ and HDNNP levels, respectively. Going to the even larger Ag_923_-ICO nanoparticles, there is a switch in the cohesive energy since now the HDNNP value is 0.007 eV/atom lower than the PBE-D3BJ result. This is also the case for Ag_923_-CUBO, where the HDNNP cohesive energy is lower by 0.003 eV/atom. In absolute terms, the total energy of Ag_923_-ICO is lower by 15.80 and 12.69 eV than Ag_923_-CUBO, as predicted by PBE-D3BJ and the HDNNP, respectively. Here, it must be pointed out that these cuboctahedral structures are not included in the HDNNP training set; yet the HDNNP still performs very well in describing the relative stability of these structures. Furthermore, when the energy of the HDNNP structures is recalculated with PBE-D3BJ, the obtained energies are very close to those corresponding to the PBE-D3BJ-optimized structures, and the energy difference between the two methods is within 0.001 eV/atom, not only for the icosahedral nanoparticles but also for the cuboctahedral nanoparticles.

The accuracy of the HDNNP is further tested by including temperature effects. To this end, molecular dynamics (MD) simulations were performed for icosahedral Cu_148_, Ag_148_, and Cu_55_@Ag_93_ at various temperatures, that is, below the melting point, near the melting point, and above the melting point. For each MD simulation, the initial structure was taken from a 1 ns run with the HDNNP, and then it continued for 20 ps using either the HDNNP or the PBE-D3BJ level, respectively. The as-obtained radial pair distribution functions (RPDFs) are summarized in Figure 3. For all of the nanoparticles at the temperatures from below the melting point to above the melting point, the shape, location, and height of the RPDFs obtained from the HDNNP are very close to the PBE-D3BJ ones, suggesting that the HDNNP can reproduce very well the properties of pure Ag and Cu and Cu–Ag bimetallic nanoparticles of realistic size in the temperature range from 0 K to high temperatures above the melting point.

**Figure 3:**
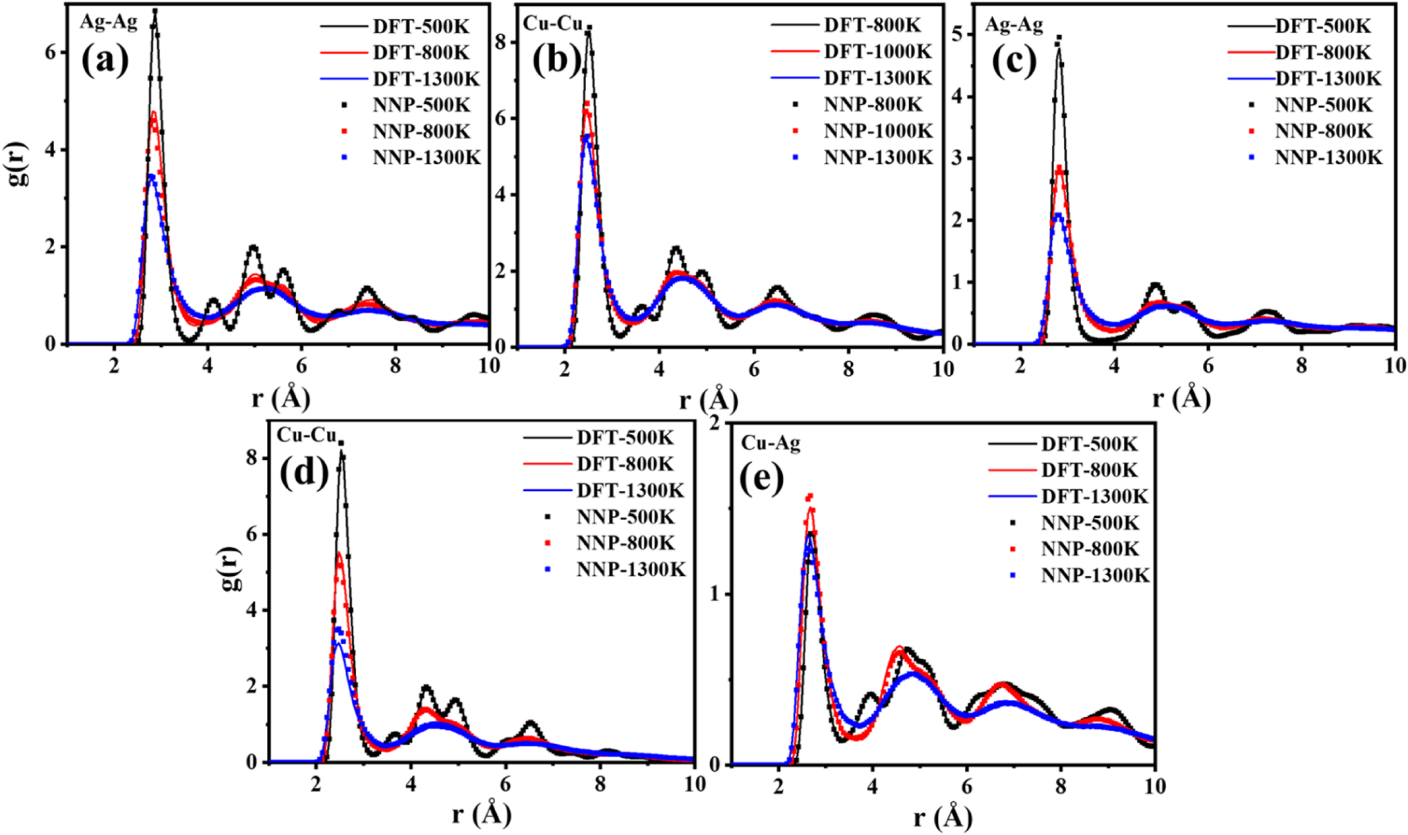
Comparison of the RPDF of Ag_148_ (a), Cu_148_ (b), and Cu_55_Ag_93_ (c–e) with C-HDNNP and DFT at different temperatures.

A high computational efficiency is also critical for the HDNNP. We tested the time consumption of molecular dynamics simulations of Ag nanoparticles (Ag_100_, Ag_300_, Ag_500_, Ag_700_, and Ag_900_) at 1000 K using the HDNNP and PBD-D3BJ, respectively. As shown in Figure S3, the computational speed of the HDNNP is more than 1000 times faster than PBE, and this performance advantage becomes even more significant when simulating larger nanoparticle systems. It should be pointed out that for odd-numbered Cu and Ag nanoparticles, the DFT time consumption should be approximately twice compared with even-numbered nanoparticles because of the unpaired electron, as spin polarization must be accounted for.

### 3.2 Global Minimum Structures of Cu and Ag Nanoparticles within 1000 Atoms

In this section, the global minimum structures of Cu and Ag nanoparticles containing 55, 147, 309, 561, and 923 atoms are considered. Besides, for each pair of nuclearities, three additional nanoparticles are considered. The minima hopping method combined with the HDNNP is used to obtain stable structures, and tens of structures with the lowest energies are reoptimized with PBE-D3BJ to determine the global minimum. As shown in Figures 4 and 5, the global minimum structures of Cu and Ag nanoparticles with 55, 147, 309, 561, and 923 atoms are icosahedral, which coincides with previous experimental and theoretical results.^35–37^ Furthermore, the Ag and Cu nanoparticles, which do not have the icosahedral magic number, can be regarded as near-icosahedral with surface adatoms with the exception of the case of quite small Cu_78_. The surface adatoms tend to gather together to maximize their coordination number. The energy of the Cu_78_ global minimum is lower than that of Cu_78_ with the Cu_55_ core by only 0.11 eV. Previous theoretical work with the PBE functional also found that Cu_201_ has icosahedral structures.^49^

**Figure 4:**
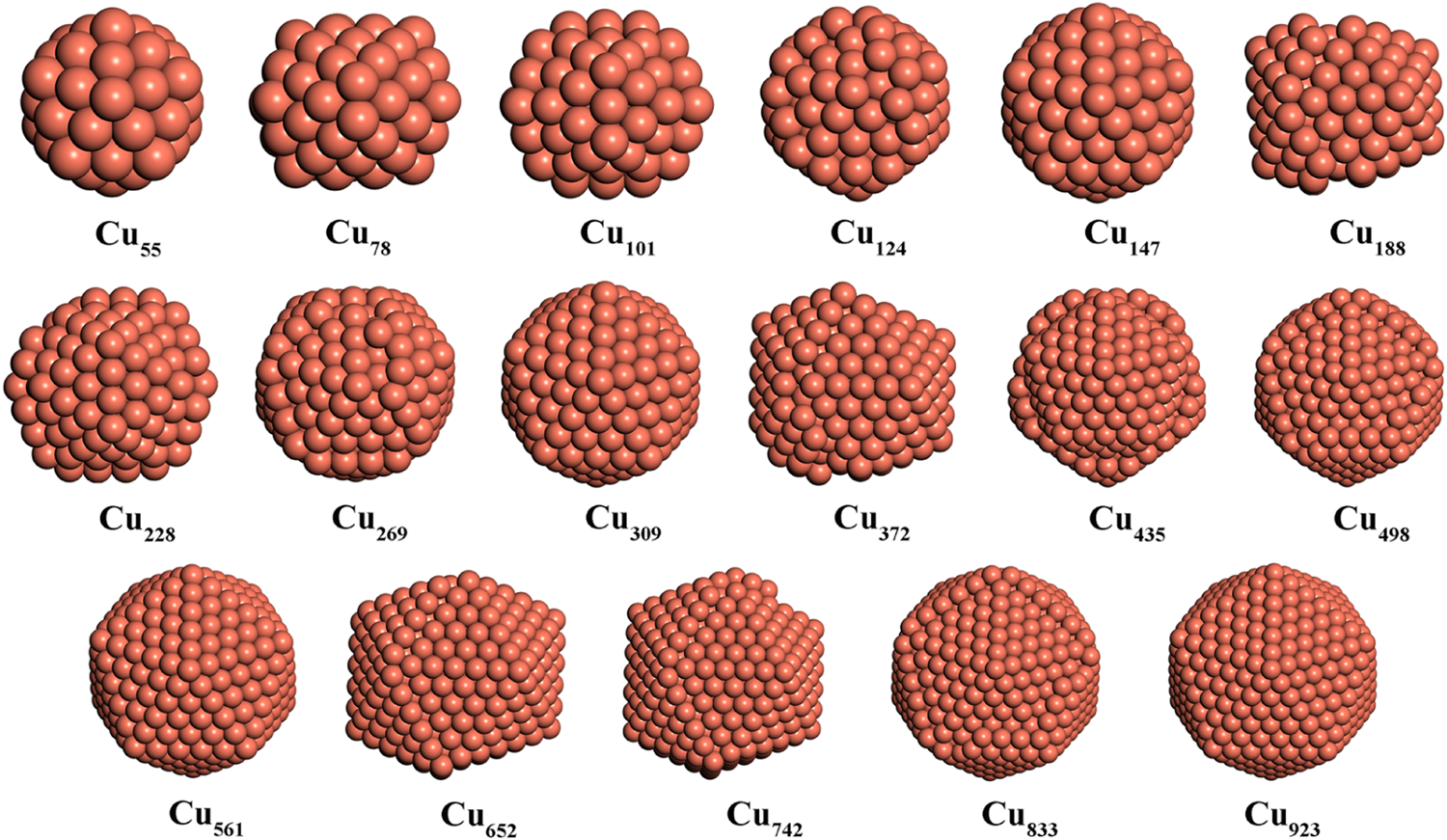
Global minimum structures of Cu_*n*_ (*n* = 55–923) (color code as in Figure 1).

**Figure 5:**
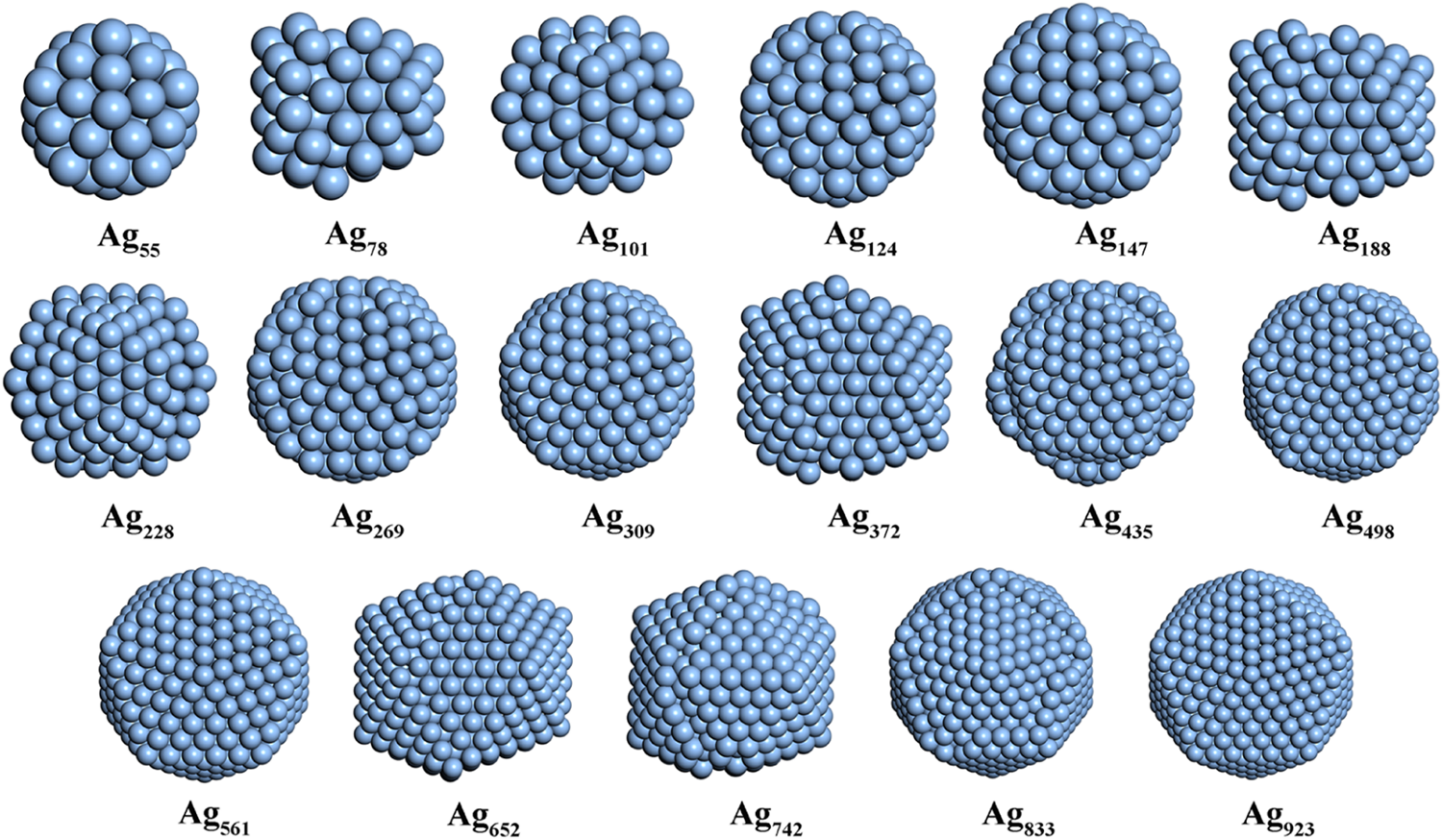
Global minimum structures of Ag_*n*_ (*n* = 55–923) (color code as in Figure 1).

Notably, Loffreda et al.^50^ recently proposed that the icosahedral Ag nanoparticles in the 309–561 atom range are metastable. However, their DFT analysis presents structural stability solely on the basis of surface energy considerations without complete total energy comparisons between different nanoparticle configurations. While the surface energy certainly contributes to the nanoparticle stability, the bulk energy term must be fully accounted for in rigorous stability assessments. The transition of Ag_191_ and Ag_700_ nanoparticles from icosahedral to decahedral was observed in molecular dynamics simulations using the Gupta potential.^51^ However, this potential can lead to results for Cu, Ag, and Au nanoparticles opposed to the ones arising from DFT, as highlighted by Ferrando et al.^49^ Taking Cu_201_ as an example, the PBE energy of the icosahedral structure is lower by 0.507 eV than that of the decahedral one, but the Gupta potential predicts the icosahedral one at a 0.9286 eV higher energy. To properly evaluate relative stabilities, we conducted global minimum structure searches for Ag_281_, Ag_282_, Ag_284_, Ag_297_, Ag_298_, Ag_301_, Ag_308_, Ag_310_, Ag_314_, Ag_318_, and Ag_321_ using both HDNNP and PBE-D3BJ methods. These structures were then compared with those from ref 50, which, for consistency, were here reoptimized using PBE-D3BJ/DZVP in CP2K. It is found that all of the most stable structures of Ag nanoparticles around 309 atoms are still icosahedral, both according to HDNNP and PBE-D3BJ methods (*cf*. Figure S4 of the SI), and their energies are much lower than those presented in the previously mentioned work (Table S2 of the SI). The excellent agreement between HDNNP and PBE-D3BJ results further validates the accuracy of the HDNNP for predicting diverse nanoparticle structures. The experimental observation of low icosahedral nanoparticle ratios in their work may be raised from their liquid nitrogen cooling protocol. Such rapid quenching to very low temperatures could kinetically trap high-temperature metastable configurations, preventing the transformation to thermodynamically stable structures. This interpretation is supported by our HDNNP molecular dynamics simulations at 300 K for 100 ps, which show that all tested nanoparticles retain their original morphologies presented in the paper. Even at elevated temperatures of 500 K for 100 ps, only Ag_281_-tCUBO, Ag_297_-tCUBO, and Ag_309_-CUBO transformed into icosahedral configurations, while others remained stable. Interestingly, other experimental studies have reported icosahedral dominance in Ag nanoparticles ranging from 1 to 6 nm.^34,53^ As the temperature was gradually decreasing, a structural transition sequence from FCC to decahedral and finally to icosahedral was observed. These results suggest that the Cu and Ag nanoparticles ranging from 100 to 1000 atoms have a very strong tendency to form icosahedral structures.

### 3.3 Structural Transition of Cu and Ag Nanoparticles

The bulk structures of Ag and Cu adopt face-centered cubic (*fcc*) lattices, making it critical to identify the threshold size at which their nanoparticles undergo the transition from icosahedral to FCC configurations. For Ag and Cu nanoparticles exhibiting FCC structures, the relative stability of octahedral, cuboctahedral, and truncated-octahedral geometries is further evaluated. According to previous experimental results,^33,34^ the decahedral structures are the intermediates for the transition of Ag and Cu nanoparticles from icosahedral to FCC. As discussed in the present work, the stability of FCC nanoparticles strongly depends on the (111) and (100) surface ratio, which may also affect the stability of decahedral nanoparticles. Due to the complexity of transition from icosahedral to decahedral and FCC with increasing size, the intermediate state of decahedral requires a systematic study which is out of the scope of the present work and will be the object of forthcoming studies where the temperature effect will also be considered. In the case of truncated-octahedral structures, the influence of having different (111) and (100) facet surface ratios has been analyzed and quantified by the ratio of two edge atoms, as shown in Figure 6. For simplicity, the edge atoms shared by two (111) surfaces are represented as *a*, and those shared by one (111) surface and one (100) surface are represented as *b*. The octahedral nanoparticles can be regarded as cuboctahedral with *b* being 0. Specifically, the present study considers *a*/*b* ratios of around 1.50, 1.00, 0.75, 0.50, and 0.33 to explore their effects on the structural stability and properties.

**Figure 6:**
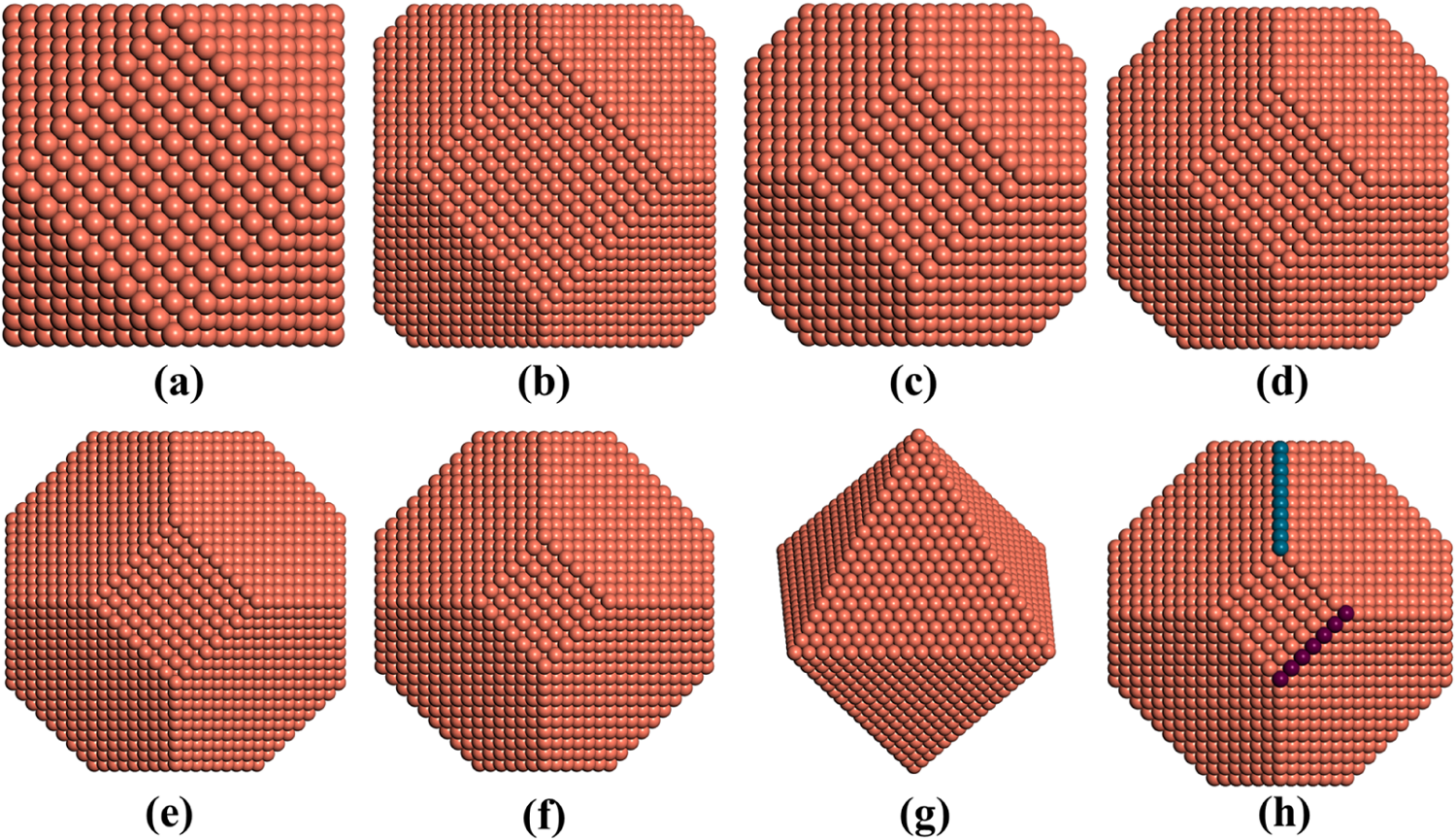
Structural models of cuboctahedral nanoparticles (a), truncated-octahedral nanoparticles with *a*/*b* ratios of 0.33 (b), 0.50 (c), 0.75 (d), 1.00 (e), and 1.50 (f), and octahedral nanoparticles (g). *a* and *b* are the edge atoms colored in dark blue and purple. The rest of the color code is the same as in Figure 1.

The scaling relationship between properties, such as the cohesive energy, with *N* ^–1/3^, where *N* is the number of atoms, has been employed in previous theoretical studies to describe the evolution of cohesive energies in Cu and Pd nanoparticles.^43,52–54^ In this study, we use this relationship to analyze the cohesive energies of the Cu and Ag nanoparticles with various shapes. Since *N* ^–1/3^ values are more widely spaced near unity, smaller nanoparticles have a larger influence on whether the relationship is fulfilled. Also, the impact of the error in the cohesive energy of the smaller nanoparticles on this relationship is greater than that of the larger nanoparticles. To address this issue, nanoparticles containing more than 1,000 atoms are used to deduce the cohesive energies of very large nanoparticles. Simultaneously, the relationship is also analyzed for nanoparticles with fewer than 1000 atoms considered for comparison purposes.

The relationship between the average cohesive energy and *N* ^–1/3^ for Cu nanoparticles with more than 1000 atoms is shown in Figure 7, displaying a very good linear relationship for all of the considered models depicted in Figure 6, with *R* ^2^ values almost equal to one. According to the fitted linear relationships, when *N* ^–1/3^ is larger than 0.0547, namely, the atom number smaller than 6102, the icosahedral nanoparticles have the highest cohesive energies. Upon increasing the nanoparticle size, the truncated-octahedral nanoparticles with the *a*/*b* ratio around 0.50 have higher cohesive energies, and this is maintained until *N* ^–1/3^ decreases to 0.0201, corresponding to the 122,613 atoms. For even larger nanoparticles, the truncated-octahedral nanoparticles with an *a*/*b* ratio around 0.33 become the most stable ones. It is noted that for very large nanoparticles, the average cohesive energy differences between the truncated-octahedral and cuboctahedral structures are very small. For example, for *N* ^–1/3^ close to zero, the *E*_coh_ values are −3.9613, −3.9618, and −3.9616 eV/atom for the truncated-octahedral nanoparticles with *a*/*b* ratios around 0.50 and 0.33 and the cuboctahedral nanoparticles, respectively. Interestingly, these values are also very close to the average cohesive energy of bulk Cu, which is −3.9624 and −3.9618 eV/atom as predicted by PBE-D3BJ and HDNNP calculations, respectively. Here, the HDNNP cannot distinguish such small differences, indicating the limit of its accuracy. On the other hand, the octahedral nanoparticles and other considered truncated-octahedral nanoparticles, where the *a*/*b* ratio is around 1.50, 1.00, and 0.75, also have higher cohesive energies than the icosahedral nanoparticles. For *N* ^–1/3^ close to 0, they are −3.9582, −3.9605, −3.9607, and −3.9609 eV/atom, respectively. Nevertheless, at this size, no nanoparticles with higher *a*/*b* ratios are found as the most stable. Thus, the relationships between the average cohesive energy and the size clearly show that Cu nanoparticles transition from icosahedral to cuboctahedral as the size increases.

**Figure 7:**
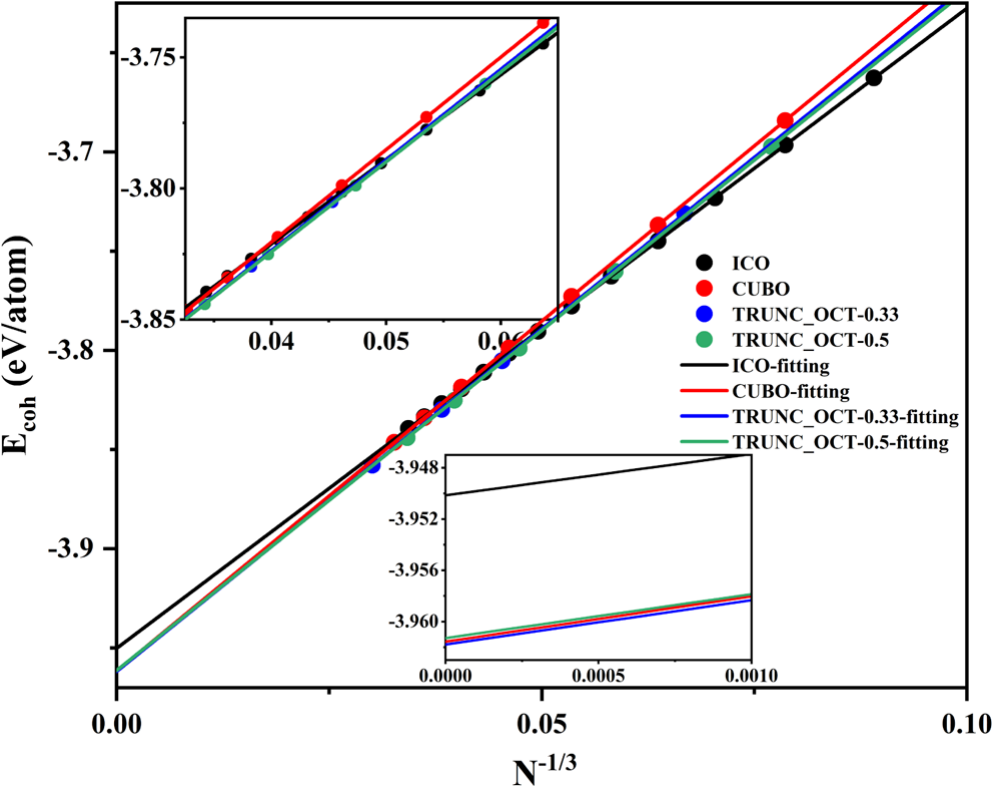
Linear relationship between the average cohesive energy and the size of Cu nanoparticles with over 1000 atoms. The insets magnify the region for the large (top) and extremely large (bottom) nanoparticles.

For smaller nanoparticles, ranging from 55 to 1000 atoms, the fitted linear relationship between the average cohesive energy and *N* ^–1/3^ is similar to that obtained by considering *b* nanoparticles larger than 1000 only (*cf*. Figure S5). The Cu nanoparticles transform from icosahedral into truncated-octahedral with the *a*/*b* ratio around 0.50 at *N* ^–1/3^ smaller than 0.0537 or N larger than 6455. When *N* ^–1/3^ decreases to 0.0224 with N increasing to 8,854, and further to 0.0110, with N becoming 741,364, they continue to transform to truncated-octahedral, with *a*/*b* around 0.33, and cuboctahedral, respectively, suggesting that the very large Cu nanoparticles prefer cuboctahedral structures. However, when *N* ^–1/3^ is close to zero, the cohesive energy of the cuboctahedral nanoparticle is −3.9628 eV/atom, which is slightly higher than that of bulk Cu.

Regarding the Ag nanoparticles, Figure 8 shows a very good linear relationship between the average cohesive energy and *N* ^–1/3^ for all of the considered Ag nanoparticles. For *N* ^–1/3^ larger than 0.0460, or less than 10,251 atoms, the icosahedral nanoparticles have the highest cohesive energies. Upon increasing the size, the truncated-octahedral nanoparticles with *a*/*b* ratios around 0.50 have higher cohesive energies, and this holds until *N* ^–1/3^ decreases to 0.0275 or 48,274 atoms. For *N* ^–1/3^ smaller than 0.0031, namely, above 32,830,996 atoms, the truncated-octahedral nanoparticles with *a*/*b* ratios around 0.33, transform into cuboctahedral nanoparticles. Hence compared to Cu, Ag nanoparticles prefer the *fcc* structure at a larger size, which nicely coincides with previous experimental results.^33,34^ For very large sizes, the average cohesive energy differences between the truncated-octahedral and cuboctahedral Ag nanoparticles are still very small. For *N* ^–1/3^ close to 0, the *E*_coh_ values are −3.0058, −3.0061, −3.0060, −3.0064, and −3.0066 eV/atom for the truncated-octahedral nanoparticles with *a*/*b* ratios of 1.50, 1.00, 0.75, 0.50, and 0.33, respectively. For icosahedral, octahedral, and cuboctahedral nanoparticles of this size, the *E*_coh_ values are −2.9990, −3.0048, and −3.0068 eV/atom, respectively. These values are close to the cohesive energy of bulk Ag, −3.0077 and −3.0061 eV/atom, according to PBE-D3BJ and HDNNP calculations, respectively. The relationships between the average cohesive energy and the size also clearly show the tendency of Ag nanoparticles to move from icosahedral to cuboctahedral structures as the size increases.

**Figure 8:**
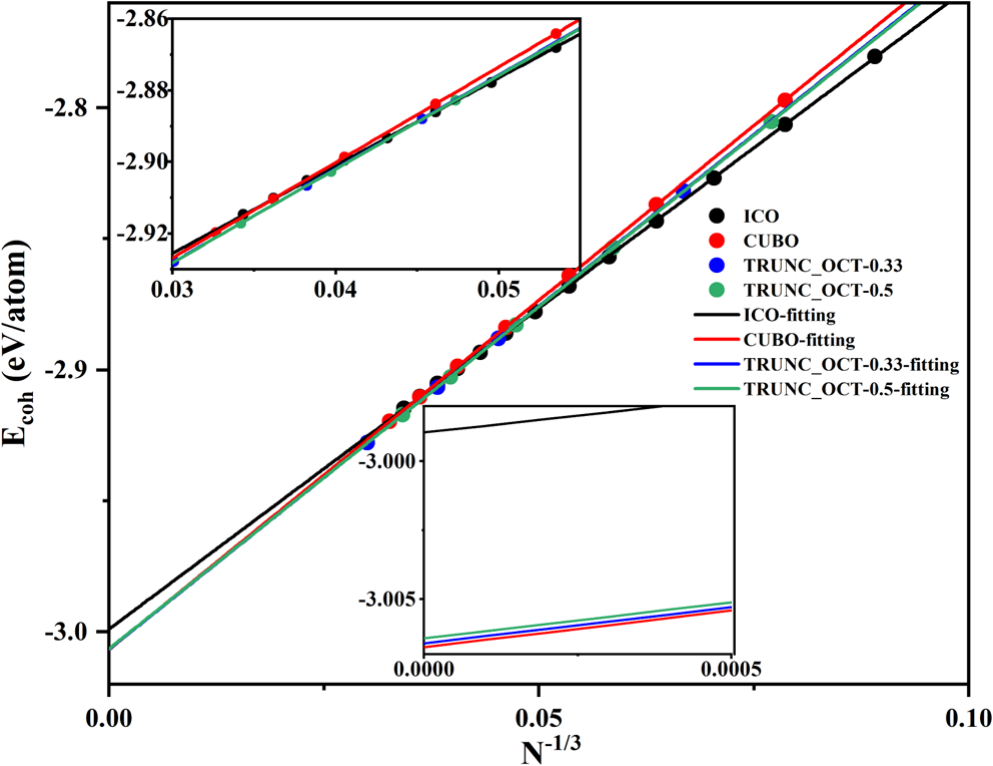
Linear relationship between the average cohesive energy and the size of Ag nanoparticles with over 1,000 atoms. The insets magnify the region for the large (top) and extremely large (bottom) nanoparticles.

The fitted linear relationship between average *E*_coh_ and *N* ^–1/3^ for Ag nanoparticles ranging from 55 to 1000 atoms is similar to that obtained by considering nanoparticles larger than 1000 atoms (*cf*. Figure S6 of the SI). At *N* ^–1/3^ smaller than 0.0383, which corresponds to N larger than 17,819, the Ag nanoparticles prefer a truncated-octahedral structure with the *a*/*b* ratio around 0.50 rather than the icosahedral structure. When *N* ^–1/3^ decreases further to 0.0303, meaning N increases to 36,055, the cuboctahedral structure continues to be the most stable one. However, for *N* ^–1/3^ close to zero, with the *a*/*b* ratio around 0.50, the *E*_coh_ of the cuboctahedral nanoparticle is −3.0097 eV/atom, which is still slightly higher than the bulk Ag value. By comparing *E*_coh_ values of cuboctahedral Cu and Ag nanoparticles to their corresponding bulk values, one finds that for extremely small *N* ^–1/3^ values, close enough to zero, the *E*_coh_ values derived from the linear relationships, where nanoparticles with only larger sizes are considered, are closer to the bulk value.

For comparison, the crossover of the preferred structure from icosahedral to cuboctahedral derived from the relationship obtained with nanoparticles smaller than 2,000 atoms is considered using data from both PBE-D3BJ and HDNNP calculations. For icosahedral and cuboctahedral nanoparticles containing 1,415 atoms, one additional atom is added to eliminate the spin effect, thus reducing the computational cost. For the considered small Cu and Ag nanoparticles, there are still very good linear relationships, as shown in Figures S3 and S6 of the SI. However, according to the PBE-D3BJ results, *E*_coh_ values for the cuboctahedral Cu and Ag nanoparticles at *N* ^–1/3^ close to 0 are −3.9731 and −3.0238 eV/atom, which are much higher than the corresponding bulk values, clearly indicating that these nanoparticles are not large enough. The preference for icosahedral Cu and Ag nanoparticles transforms into the preference for cuboctahedral nanoparticles for *N* ^–1/3^ = 0.0272 (*N* = 49,693) and 0.0205 (*N* = 116,075), respectively. According to the HDNNP calculations (see Figures S4 and S7 of the SI), *E*_coh_ values for the cuboctahedral Cu and Ag nanoparticles at *N* ^–1/3^ close to zero are −3.9646 and −3.0134 eV/atom, which are lower by 0.0085 and 0.0104 eV/atom than the PBE-D3BJ values, respectively. The *E*_coh_ extreme values for the icosahedral nanoparticles are also underestimated by 0.0096 and 0.0125 eV/atom, respectively. The preference for icosahedral Cu and Ag nanoparticles changes to cuboctahedral for *N* ^–1/3^ = 0.0330 (*N* = 27605) and 0.0310 (*N* = 33283), respectively. The *N* ^–1/3^ crossover values predicted by the HDNNP are very close to those predicted by PBE-D3BJ. But the nanoparticle nuclearity at which the crossover occurs differs significantly; this is simply because for *N* ^–1/3^ values close to 0, a small difference leads to a remarkable change of the number of atoms. Furthermore, we also investigated the effect of dispersion on the Cu and Ag structural transition. When this is not taken into account (PBE instead of PBE-D3BJ), the change of preference from icosahedral Cu and Ag nanoparticles into cuboctahedral occurs at *N* ^–1/3^ = 0.0375 (*N* = 19035) and 0.0486 (*N* = 8712), respectively (*cf*. Figures S5 and S8 of the SI). The nuclearity values are much smaller than those predicted by PBE-D3BJ, suggesting that a weaker binding strength implies that the relative stability of icosahedral Cu and Ag nanoparticles with respect to *fcc* structures occurs at smaller sizes.

In order to explain the change in the preferred structure of Cu and Ag nanoparticles for a large enough size, we analyze the radial pair distribution function (RPDF). Figure 9 shows that all RPDF peaks of icosahedral Cu and Ag nanoparticles are broader than those of cuboctahedral nanoparticles, which have more intense RPDF peaks. Besides, the shape of RPDF peaks for icosahedral nanoparticles changes remarkably as the size increases from 923 to 6,525 atoms, and the RPDF peaks for *r* > 6 Å become very broad. On the contrary, the shape and location of RPDF peaks of cuboctahedral nanoparticles do not depend on the nanoparticle size, except for a very small peak at 2.40 Å, which nearly disappears due to the significant decrease of the surface ratio as the size increases. It is noted that there are also very good linear relationships between the average coordination number and *N* ^–1/3^, as shown in Figure S13 of the SI. For all of the considered nanoparticles, the icosahedral nanoparticles have the largest average coordination number, and it decreases with the decreasing *a*/*b* ratio for the truncated-octahedral nanoparticles. For *N* ^–1/3^ values close to zero, the average coordination number increases to 12 for all of the different nanoparticles. The average bond length of the icosahedral Cu nanoparticles increases from 2.516 Å for Cu_55_ to 2.538 Å for Cu_561_ and then remains constant (2.535 Å) for nanoparticles larger than Cu_14993_ (see Figure 10). The large cuboctahedral and truncated-octahedral Cu nanoparticles have almost the same average bond length, which is 0.006 Å shorter than that of icosahedral nanoparticles. Similarly, the average bond length of the icosahedral Ag nanoparticles increases from 2.876 Å for Ag_55_ to 2.895 Å for Ag_561_ and remains constant (2.894 Å) for nanoparticles larger than Ag_6525_. The large cuboctahedral and truncated-octahedral Ag nanoparticles still have almost the same average bond length, which is 0.003 Å shorter than that of icosahedral nanoparticles.

**Figure 9:**
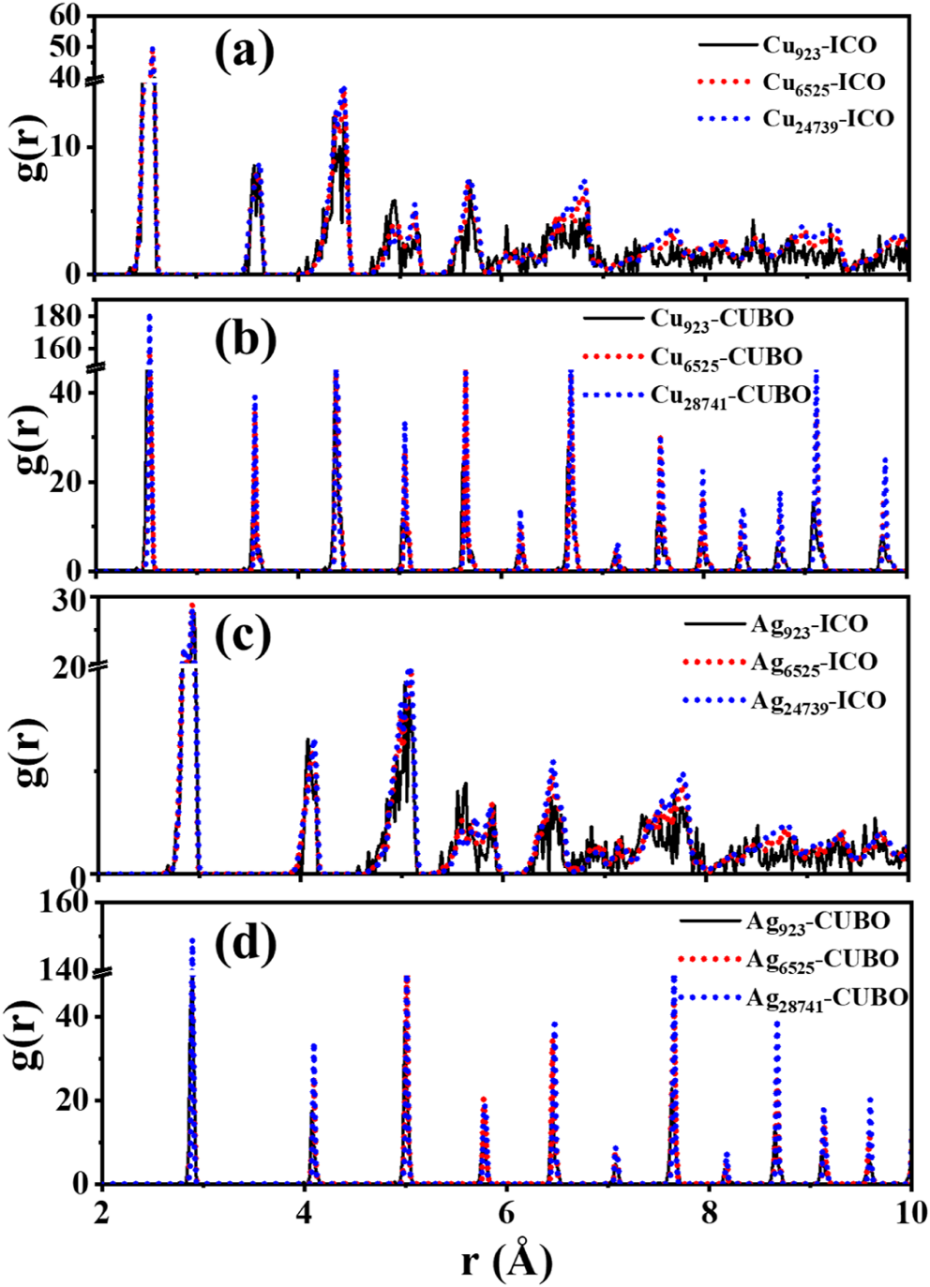
RPDF of icosahedral and cuboctahedral Cu (a, b) and Ag (c, d) nanoparticles.

**Figure 10:**
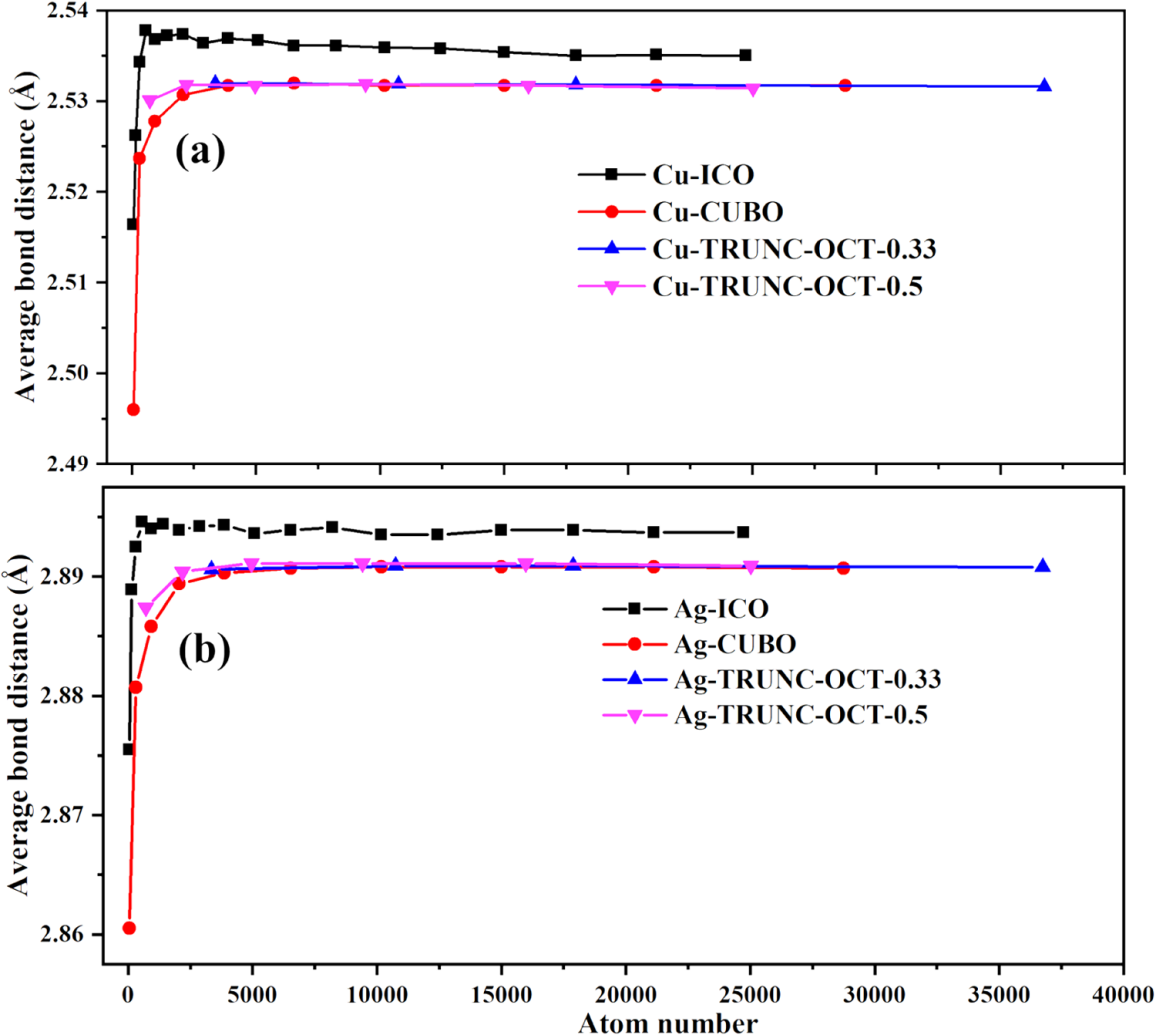
Average bond length of Cu (a) and Ag (b) nanoparticles.

## 4 Conclusions

Using a large amount of training data generated through the combination with the minima hopping method and DFT calculations, HDNNPs of Cu, Ag, and Cu–Ag bimetallic particles including CO adsorption were trained and applied here to investigate the structure of Cu and Ag nanoparticles up to millions of atoms. Bimetallic nanoparticles and the interaction of Cu, Ag, and Cu–Ag nanoparticles with CO will be presented in a subsequent work.

For the Cu and Ag nanoparticles of sizes that are affordable to DFT calculations, the HDNNP accuracy is comparable to that of PBE-D3BJ. Interestingly, the HDNNP can reproduce results for nanoparticles with different shapes that were not included in the training data. A clear advantage of the HDNNP is that it can also be used to simulate the properties at temperatures from 0 K to above the melting point.

Global minimum optimization for Cu and Ag nanoparticles ranging from 100 to 1000 atoms leads to an icosahedral structure for nanoparticles having magic numbers and to a structure with an icosahedral core for the rest. For larger nanoparticles, icosahedral, octahedral, cuboctahedral, and truncated-octahedral shapes have been studied, and for the latter, various (100)/(111) surface ratios were considered. On increasing the size to more than 6,000 and 10,000 atoms for Cu and Ag nanoparticles, respectively, the structural preference for Cu and Ag nanoparticles changes from icosahedral to truncated-octahedral. For these nanoparticles, the truncated-octahedral structures with a lower (100)/(111) surface ratio become more stable, and for even larger nanoparticles, the cuboctahedral structure becomes the most stable. The preference for *fcc* structures for large nanoparticles can be attributed to their shorter and more uniform bond lengths. Also, in determining the structural preference, the bulk energy is more important than the surface energy, and this is why cuboctahedral nanoparticles become the most stable for very large nanoparticles.

For the nanoparticles with a similar shape, a very good linear relationship between the average cohesive energy and *N* ^–1/3^ is found. For the very large cuboctahedral nanoparticles with *N* ^–1/3^ close to zero, the fitted cohesive energy is very close to the bulk value. For the nanoparticles with a similar shape, there is also a very good linear relationship between the average coordination number and *N* ^–1/3^, with the icosahedral structures having a higher average coordination number than the other. For nanoparticles ranging from hundreds to thousands of atoms, the average coordination number dominates the stability. Besides, in the limit of very large particles with *N* ^–1/3^ approaching zero, the average coordination number of all nanoparticles nicely converges to 12, which is the bulk value.

To summarize, the developed HDNNP allows examining the properties of Cu and Ag nanoparticles containing several thousand atoms at DFT (here, PBE-D3BJ) accuracy at an affordable computational cost.

## Supplementary Material



## Notes

Energy and force root-mean-square error of eight high-dimensional neural network potentials with different random seeds (Table S1); relative energy of Ag nanoparticles with FCC and decahedral structures reported in ref 50 of the main text, relative to the global minimum structures obtained in this work (Table S2); number of atoms of the different nanoparticles calculated using HDNNP (Table S3); parameters of linear relationships *y = ax + b* and structure crossover values for Cu and Ag nanoparticles of sizes above or below 1000 atoms (Tables S4–S7); parameters of linear relationships *y = ax + b* between the average coordination number and the nanocluster size (Table S8); snapshots of Ag_310_ and Ag(111), respectively, during minima hopping simulation (Figures S1 and S2); comparison of time consumption for calculations with HDNNP and PBE (Figure S3); global minimum structures of Ag nanoparticles (Figure S4); linear relationship between the average cohesive energy and the size of Cu and Ag nanoparticles larger than 55 atoms (Figures S5 and S6); linear relationship between the average cohesive energy computed with different methods and the size of Cu and Ag nanoparticles ranging from 55 to 1416 atoms (Figures S7–S12); and linear relationship between the average coordination number and the nanocluster size (Figure S13) (PDF)
