# Correction to “Machine Learning Potential Analysis of Structural Transition in Cu and Ag Nanoparticles: From Icosahedral to Face-Centered Cubic”

**DOI:** 10.1021/acs.jctc.5c01651

**Published:** 2025-10-21

**Authors:** Yongpeng Yang, Jingli Han, Francesc Viñes, Francesc Illas

According to the specific policies of the University of Barcelona, this affiliation is not permitted for use in publications by visiting scholars. Yongpeng Yang was unaware of this policy at the time of submission. The affiliation of Yongpeng Yang should be the primary institution: Henan Institute of Advanced Technology, Zhengzhou University, Zhengzhou, 450052, P. R. China. The affiliation for the University of Barcelona should be removed from Yongpeng Yang’s name.

