# Supplementary material for: Machine Learning Potential Analysis of Structural Transition in Cu and Ag Nanoparticles: From Icosahedral to Face-Centered Cubic
Source: J Chem Theory Comput. 2025 Aug 19;21(17):8601–13. doi: 10.1021/acs.jctc.5c00791 (PMC12424160; doi:10.1021/acs.jctc.5c00791)
Supplement: Supplementary file 1 [file ct5c00791_si_001.pdf]

Machine Learning Potential Analysis of Structural Transition in Cu and Ag Nanoparticles: From Icosahedral to Face-Centered Cubic

Yongpeng Yang[a,b*], Jingli Han[c], Francesc Viñes [b], Francesc Illas[b*]

[a]*Henan Institute of Advanced Technology, Zhengzhou University, Zhengzhou 450003, China*

[b]*Departament de Ciència de Materials i Química Física & Institut de Química Teòrica i Computacional (IQTCUB), Universitat de Barcelona, c/ Martí i Franquès 1-11, 08028, Barcelona, Spain*

[c]*School of Material and Chemical Engineering, Zhengzhou University of Light Industry, Zhengzhou 450001, China*

Corresponding authors e-mail: francesc.illas@ub.edu , ypyang2017@zzu.edu.cn

Table S1. Energy and force root mean square error (RMSE) of eight high dimensional neural network potentials (HDNNP) with different random seed.

	**Energy RMSE (meV/atom)**		**Force RMSE (meV/Å)**	
	**Training set**	**Test set**	**Training set**	**Test set**
**1**	1.52	1.59	85.3	84.5
**2**	1.52	1.50	85.5	86.7
**3**	1.60	1.61	85.4	82.6
**4**	1.54	1.52	83.7	96.5
**5**	1.54	1.62	85.8	86.8
**6**	1.48	1.49	88.0	81.7
**7**	1.49	1.49	84.4	81.6
**8**	1.56	1.60	92.7	90.0

Table S2. Relative energy of Ag nanoparticles with FCC and decahedral structures reported by Loffreda *et al.* (*cf.* reference 50 in the main text) relative to the global minimum structures obtained in this work.

	**Ag_{281}-tcubo**	**Ag_{282}-marks**	**Ag_{284}-marks**	**Ag_{297}-tcubo**	**Ag_{298}-dto**	**Ag_{301}-dmarks**	**Ag_{308}-dmarks**
**PBE-D3BJ**	6.31	9.11	3.21	4.94	4.81	4.13	2.74
**NNP**	3.72	11.68	0.56	3.30	4.24	2.83	2.87

	**Ag_{309}-ino**	**Ag_{309}-cubo**	**Ag_{309}-dto**	**Ag_{310}-dmarks**	**Ag_{314}-ito**	**Ag_{314}-dmarks**	**Ag_{318}-marks**	**Ag_{321}-tcubo**
**PBE-D3BJ**	5.49	7.75	5.25	6.37	4.32	2.60	1.84	6.46
**NNP**	4.79	6.57	5.66	6.49	4.52	2.50	1.15	4.60

Table S3. Number of atoms of the different nanoparticles calculated using HDNNP. The values in the parentheses are $a*b$ for truncate-octahedral nanoparticles.

Nanocluster shape	Number of Atoms
**Icosahedral**	55, 147, 309, 561, 923, 1415, 2057, 2869, 3871, 5083,<br>6525, 8217, 10179, 12431, 14993, 17885, 21127, 24739, 28741
**Cuboctahedral**	55, 309, 923, 2057, 3871, 6525, 10179, 14993, 21127, 28741
**TRUNC-CUB-0.33**	3355 (3*9), 10744 (4*13), 17879 (5*15), 36752 (6*19)
**TRUNC-CUB-0.5**	711(3*5), 2190(4*7), 4957(5*9), 9416(6*11), 15971 (7*13), 25026(8*15)
**TRUNC-CUB-0.75**	976(4*5), 2735(5*7), 6895(7*9), 12334(8*11), 22314 (10*13)
**TRUNC-CUB-1.0**	201(3*3), 1289(5*5), 4033 (7*7), 9201 (9*9), 17561(11*11), 29881(13*13)
**TRUNC-CUB-1.5**	314(4*3), 2075(7*5), 6560(10*7), 15045(13*9), 28806 (16*11)
**Octahedral**	85, 231, 489, 891, 1469, 2255, 3281, 4579, 6181, 8119, 104251

Table S4. Parameters of linear relationships $y = ax + b$ and crossover values between each two linear relationships of Cu nanoparticles larger than 1,000 atoms.

	a	***b***	R^2	**Cross-ICO**	**Cross-CUBO**	**Cross-0.33**	**Cross-0.5**	**Cross-0.75**	**Cross-1.0**	**Cross-1.5**
**Icosahedral**	3.2258	-3.9502	1.00000							
**Cuboctahedral**	3.5282	-3.9616	1.00000	0.0377						
**TRUNC-CUB-0.33**	3.4542	-3.9618	1.00000	0.0510	-0.0031					
**TRUNC-CUB-0.50**	3.4294	-3.9613	1.00000	0.0547	0.0027	0.020129				
**TRUNC-CUB-0.75**	3.42629	-3.9609	1.00000	0.0538	0.0062	0.0308	0.1158			
**TRUNC-CUB-1.00**	3.43496	-3.9607	1.00000	0.0505	0.0090	0.0555	-0.1026	-0.0242		
**TRUNC-CUB-1.50**	3.46062	-3.9605	1.00000	0.0440	0.0158	-0.2038	-0.0256	-0.0128	-0.0090	
**Octahedral**	3.60607	-3.9582	0.99999	0.0211	-0.0433	-0.0237	-0.0175	-0.0152	-0.0148	-0.0158

Table S5. Parameters of linear relationships $y = ax + b$ and crossover values between each two linear relationships of Cu nanoparticles with size less than 1,000 atoms included.

	a	*b*	R^2	Cross-ICO	Cross-CUBO	Cross-0.33	Cross-0.5	Cross-0.75	Cross-1.0	Cross-1.5
**Icosahedral**	3.2662	-3.9525	0.99997							
**Cuboctahedral**	3.5493	-3.9628	0.99999	0.0364						
**TRUNC-CUB-0.33**	3.4542	-3.9618	1.00000	0.0492	0.0110					
**TRUNC-CUB-0.50**	3.4270	-3.9612	1.00000	0.0537	0.0136	0.0224				
**TRUNC-CUB-0.75**	3.4257	-3.9609	1.00000	0.0525	0.0156	0.0308	0.2045			
**TRUNC-CUB-1.00**	3.4243	-3.9602	1.00000	0.0483	0.0213	0.0538	0.3663	0.5177		
**TRUNC-CUB-1.50**	3.4405	-3.9595	0.99999	0.0401	0.0304	0.1641	-0.1227	-0.0934	-0.0402	
**Octahedral**	3.5407	-3.9538	0.99998	0.0047	1.0551	-0.0919	-0.0645	-0.0615	-0.0544	-0.0567

Table S6. Parameters of linear relationships y = ax + b and crossover values between each two linear relationships of Ag nanoparticles larger than 1,000 atoms.

	a	***b***	R^2	**Cross-ICO**	**Cross-CUBO**	**Cross-0.33**	**Cross-0.5**	**Cross-0.75**	**Cross-1.0**	**Cross-1.5**
**Icosahedral**	2.4489	-2.9990	0.99997							
**Cuboctahedral**	2.6656	-3.0068	1.00000	0.0360						
**TRUNC-CUB-0.33**	2.6176	-3.0066	1.00000	0.0454	0.0031					
**TRUNC-CUB-0.50**	2.6114	-3.0064	1.00000	0.0460	0.0059	0.0275				
**TRUNC-CUB-0.75**	2.6109	-3.0060	1.00000	0.0435	0.0137	0.0904	0.9556			
**TRUNC-CUB-1.00**	2.6282	-3.0061	1.00000	0.0398	0.0179	-0.0492	-0.0209	0.0046		
**TRUNC-CUB-1.50**	2.6512	-3.0058	1.00000	0.033915	0.0651	-0.0235	-0.0156	-0.0047	-0.0117	
**Octahedral**	2.7933	-3.0048	1.00000	0.0171	-0.0150	-0.0100	-0.0087	-0.0064	-0.0075	-0.0068

Table S7. Parameters of linear relationships *y*= *ax* + *b* and crossover values between each two linear relationships of Ag nanoparticles with size less than 1,000 atoms included.

	a	***b***	R^2	**Cross-ICO**	**Cross-CUBO**	**Cross-0.33**	**Cross-0.5**	**Cross-0.75**	**Cross-1.0**	**Cross-1.5**
**Icosahedral**	2.5193	-3.0029	0.99991							
**Cuboctahedral**	2.7174	-3.0097	0.99994	0.0342						
**TRUNC-CUB-0.33**	2.6176	-3.0066	1.00000	0.0372	0.0312					
**TRUNC-CUB-0.50**	2.6156	-3.0066	1.00000	0.0383	0.0303	-0.0154				
**TRUNC-CUB-0.75**	2.6188	-3.0064	1.00000	0.0344	0.0339	-0.1840	-0.0813			
**TRUNC-CUB-1.00**	2.63174	-3.0063	1.00000	0.0295	0.0403	-0.0240	-0.0230	-0.0085		
**TRUNC-CUB-1.50**	2.6476	-3.0056	1.00000	0.0210	0.0583	-0.0320	-0.0310	-0.0254	-0.0390	
**Octahedral**	2.7708	-3.0033	0.99703	0.0016	-0.1194	-0.0213	-0.0212	-0.0199	-0.0210	-0.0187

Table S8. Parameters of linear relationships $y = ax + b$ between average coordination number and nanocluster size.

	a	***b***	R^2
**Icosahedral**	-13.4160	11.9990	1.00000
**Cuboctahedral**	-16.0813	11.9983	1.00000
**TRUNC-CUB-0.33**	-15.5143	12.0025	0.99999
**TRUNC-CUB-0.50**	-15.2243	11.9997	1.00000

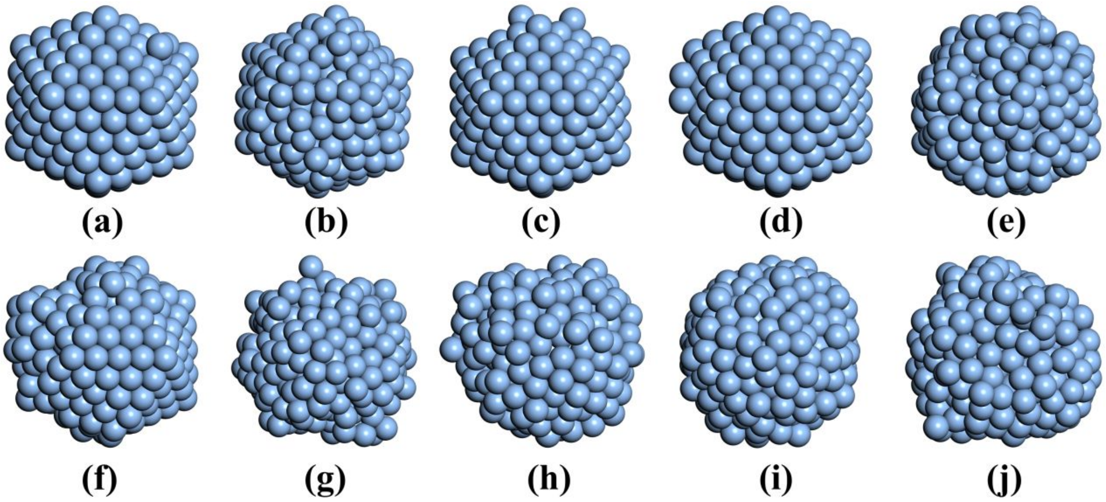

Figure S1. Snapshots of Ag_{310} during minima hopping simulation. (a) and (j) correspond to the initial and last structure, respectively.

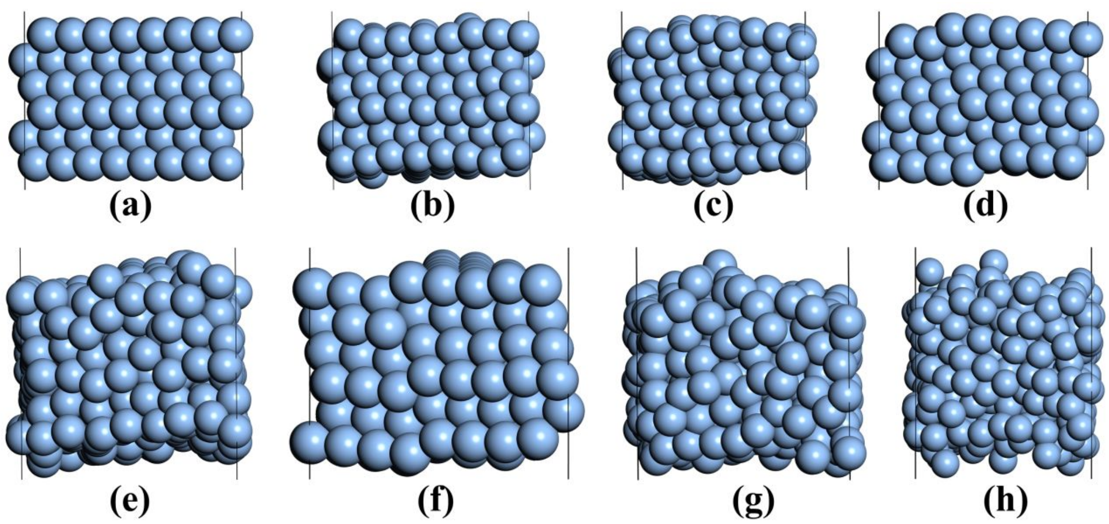

Figure S2. Snapshots of Ag(111) slab during minima hopping simulation. (a) and (h) correspond to the initial and last structure, respectively.

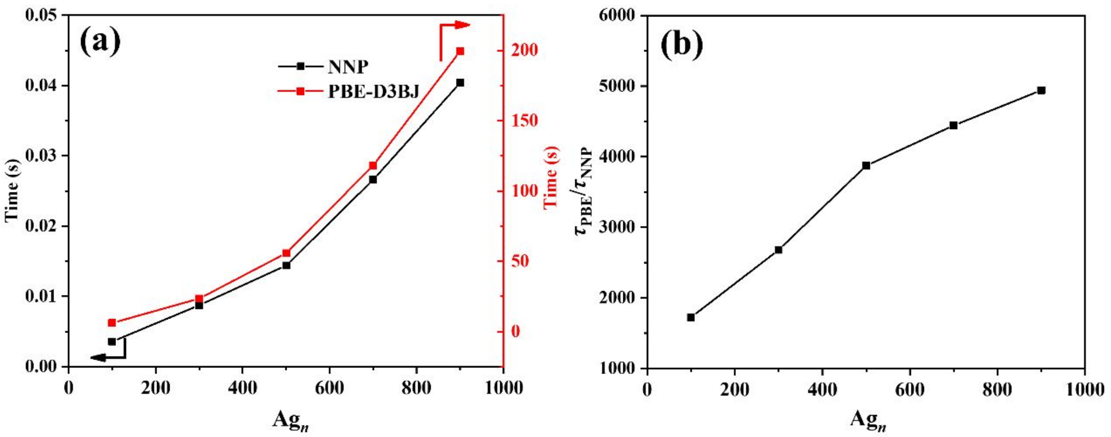

Figure S3. Comparison of time consumption for HDNNP and PBE. The tests were based on the molecular dynamic simulations of Ag nanoparticles (Ag_{100}, Ag_{300}, Ag_{500}, Ag_{700}, and Ag_{900}) at 1,000 K, and using 128 CPU cores of AMD 9554 for each calculation.

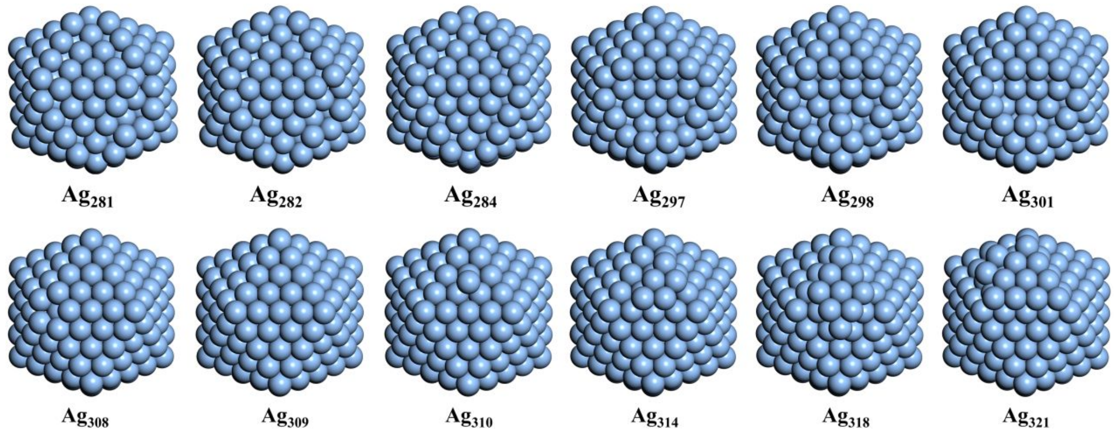

Figure S4. Global minimum structures Ag nanoparticles.

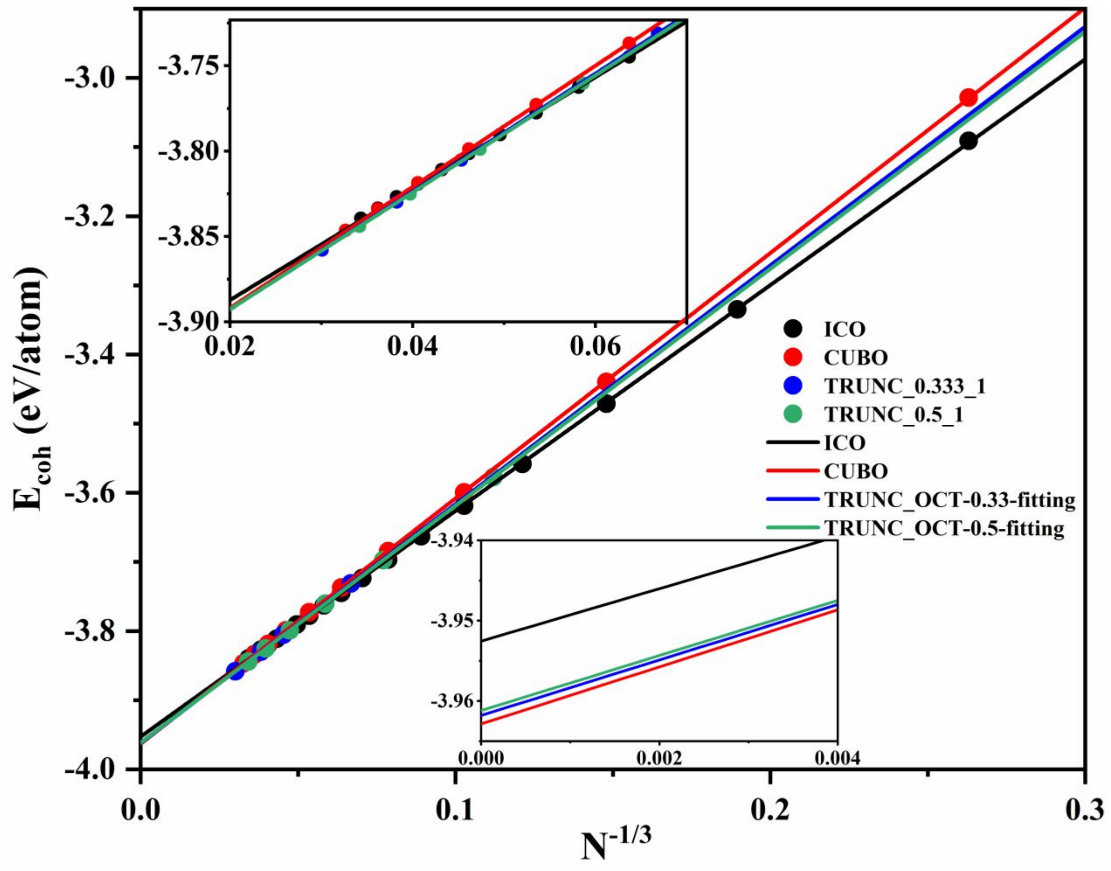

Figure S5. Linear relationship between average cohesive energy and size of Cu nanoparticles larger than 55 atoms.

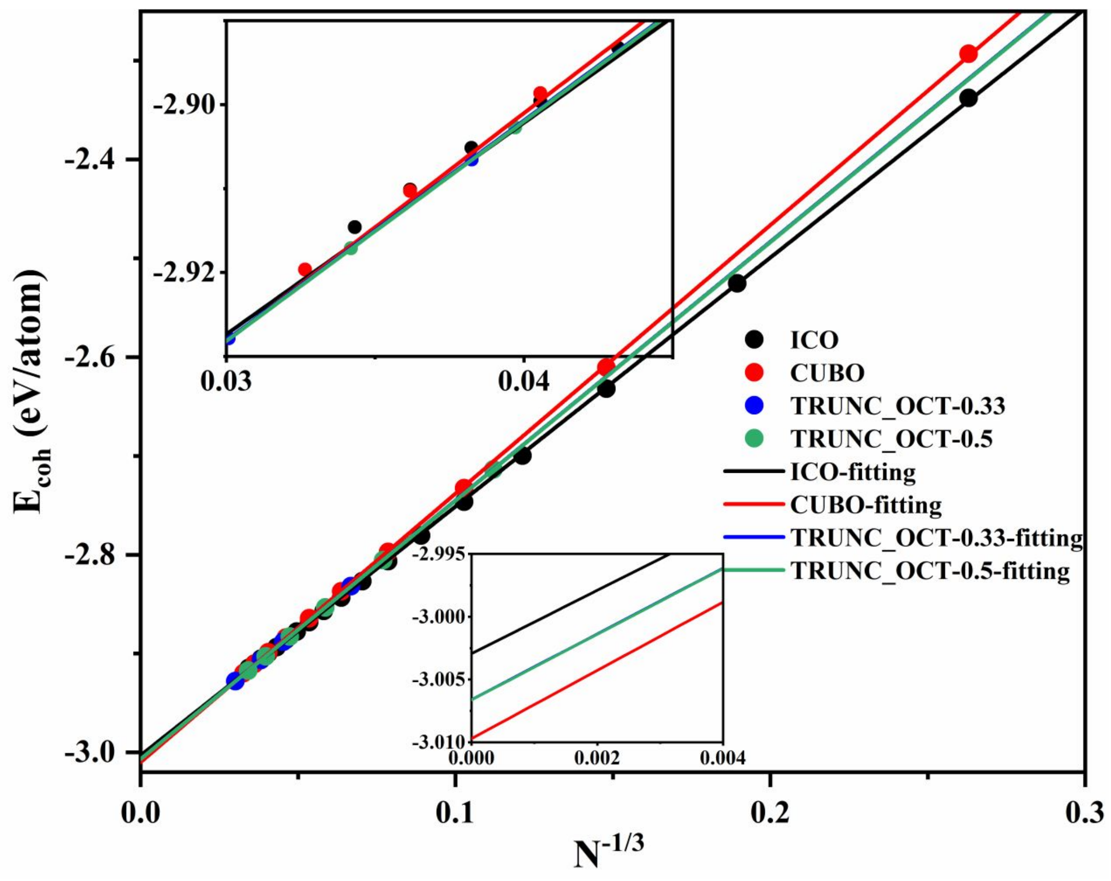

Figure S6. Linear relationship between average cohesive energy and size of Ag nanoparticles larger than 55 atoms.

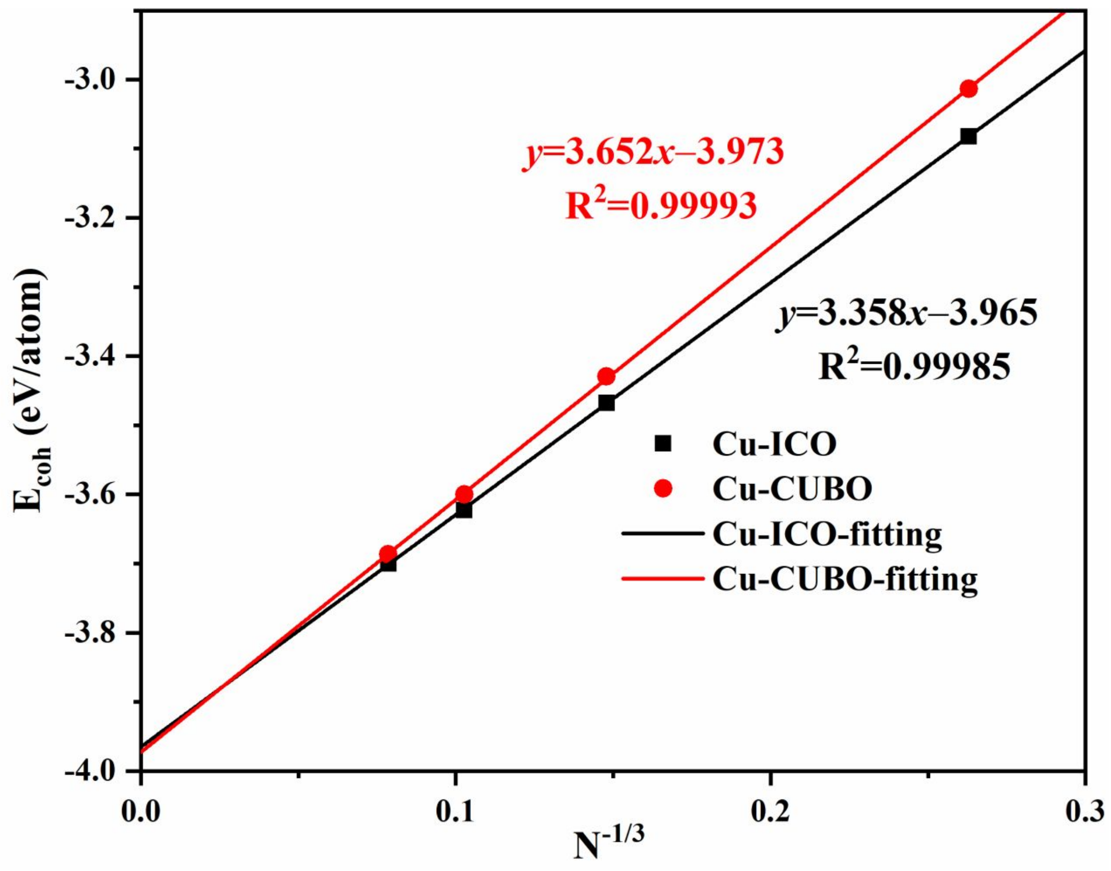

Figure S7. Linear relationship between PBE-D3BJ average cohesive energy and size of Cu nanoparticles ranging from 55 to 1,416.

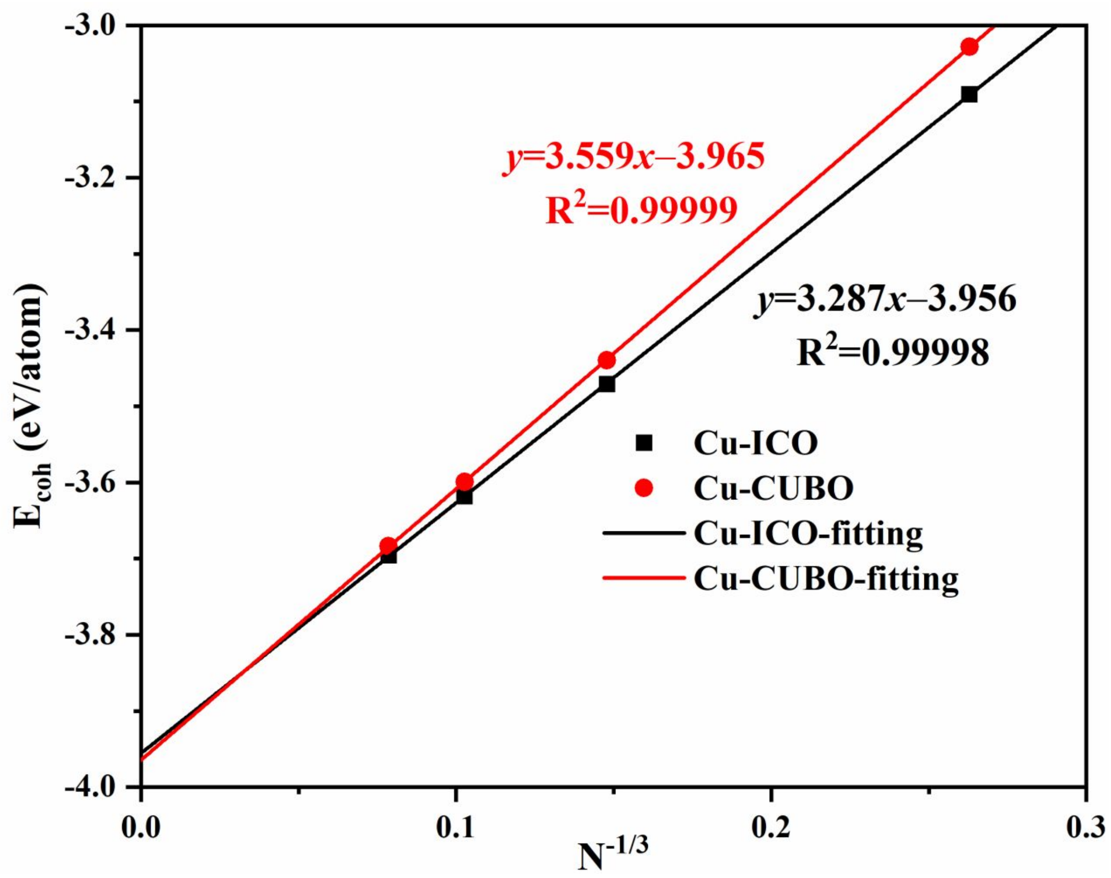

Figure S8. Linear relationship between HDNNP average cohesive energy and size of Cu nanoparticles ranging from 55 to 1,416.

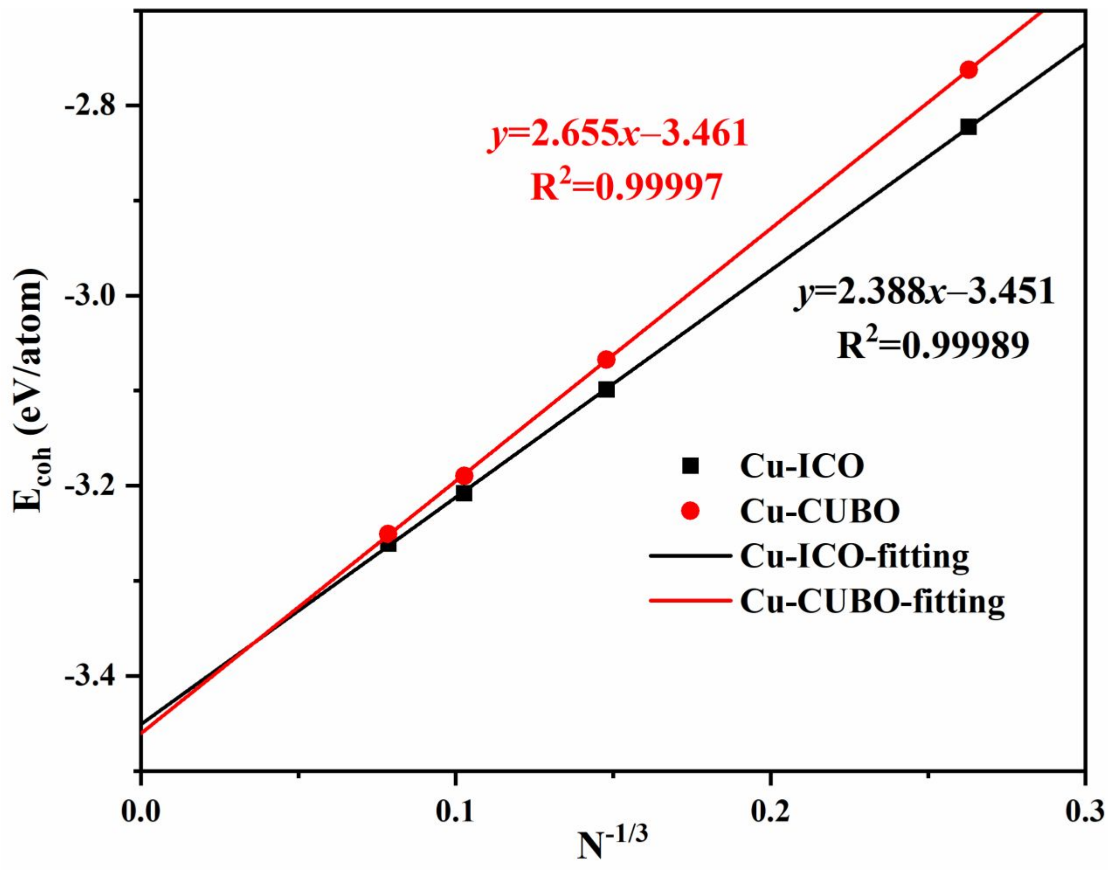

Figure S9. Linear relationship between PBE average cohesive energy and size of Cu nanoparticles ranging from 55 to 1,416.

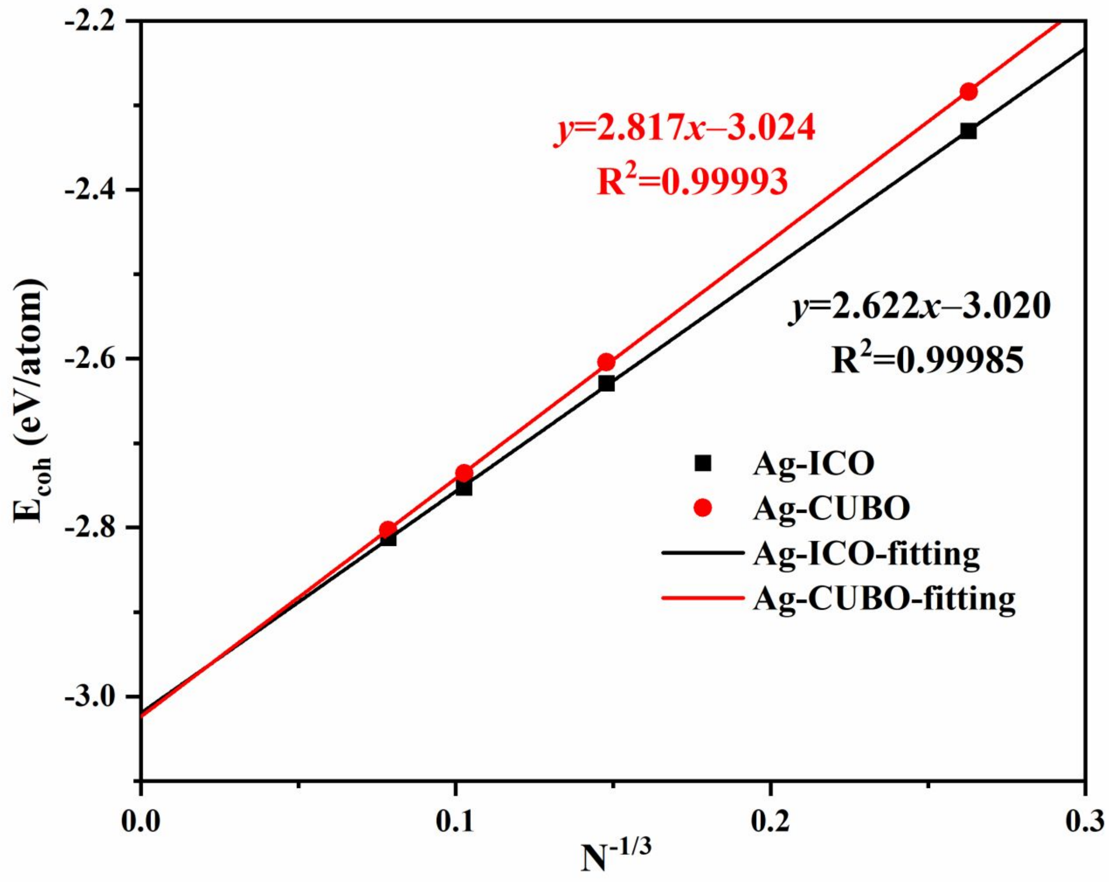

Figure S10. Linear relationship between PBE-D3BJ average cohesive energy and size of Ag nanoparticles ranging from 55 to 1,416.

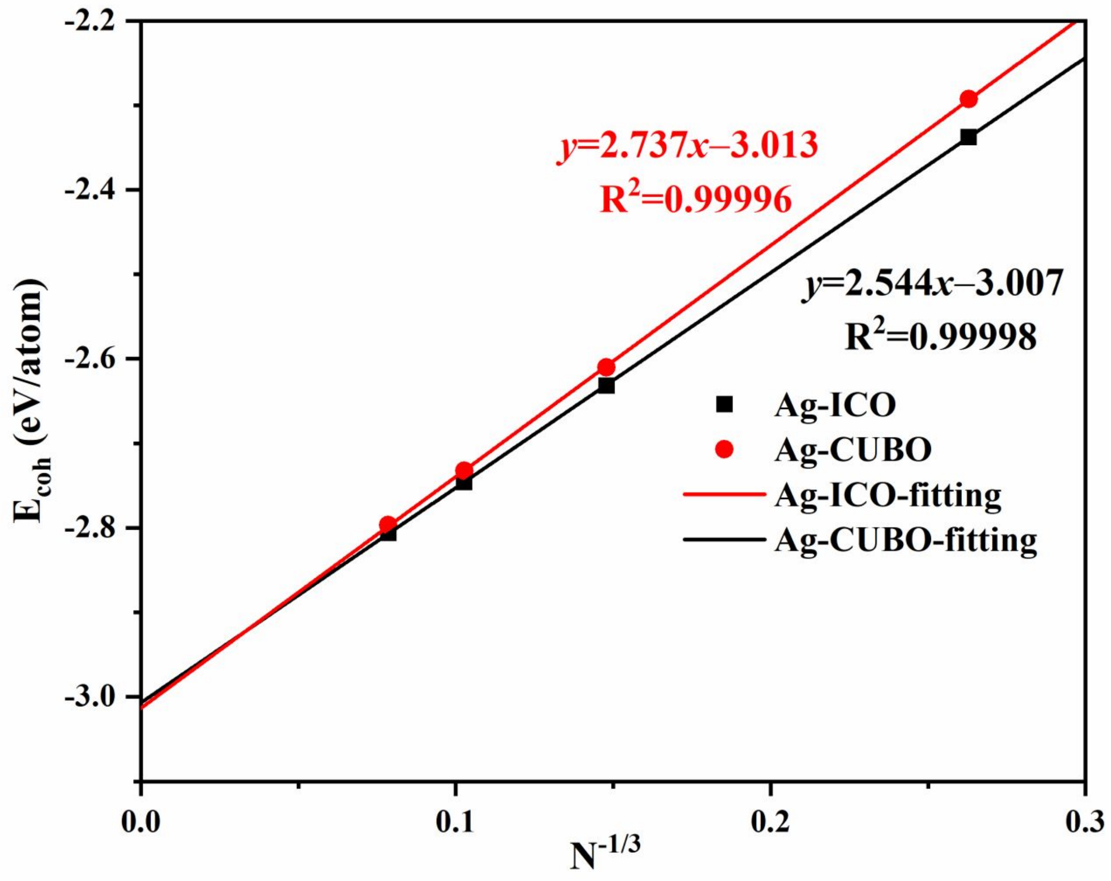

Figure S11. Linear relationship between HDNNP average cohesive energy and size of Ag nanoparticles ranging from 55 to 1,416.

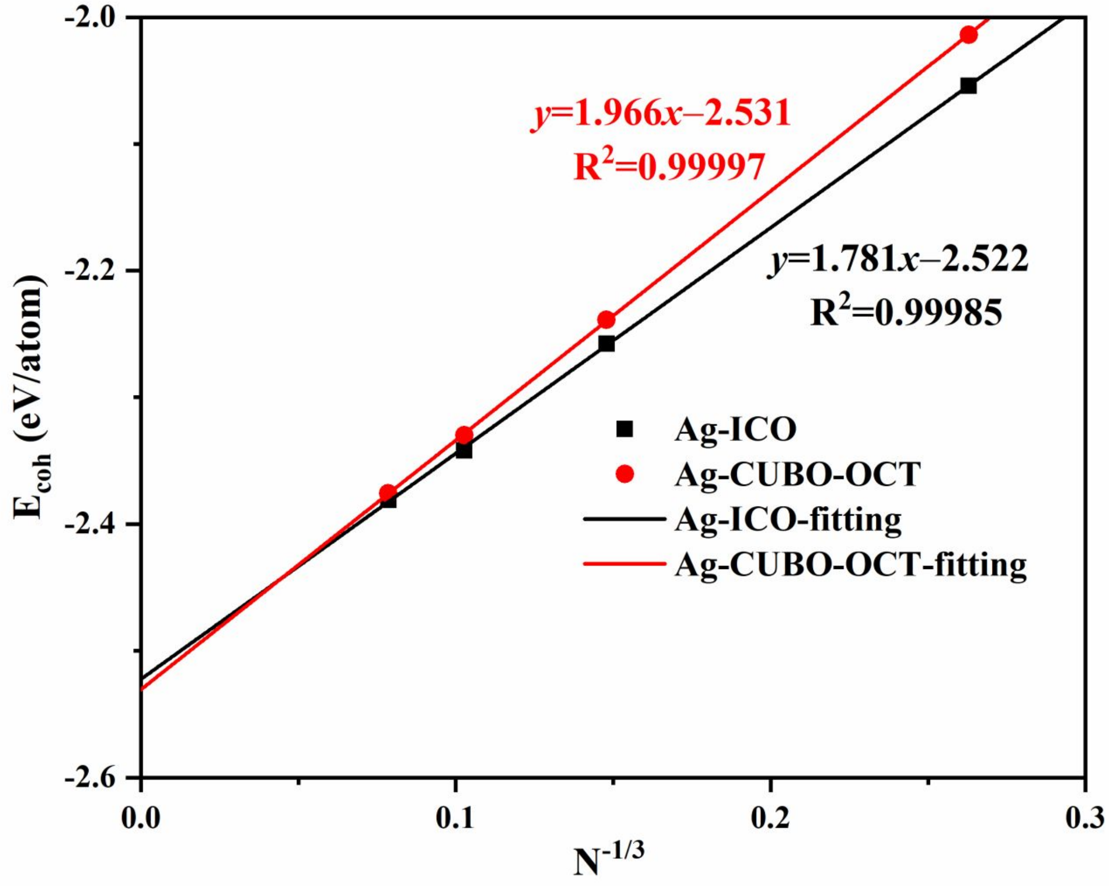

Figure S12. Linear relationship between PBE average cohesive energy and size of Ag nanoparticles ranging from 55 to 1,416.

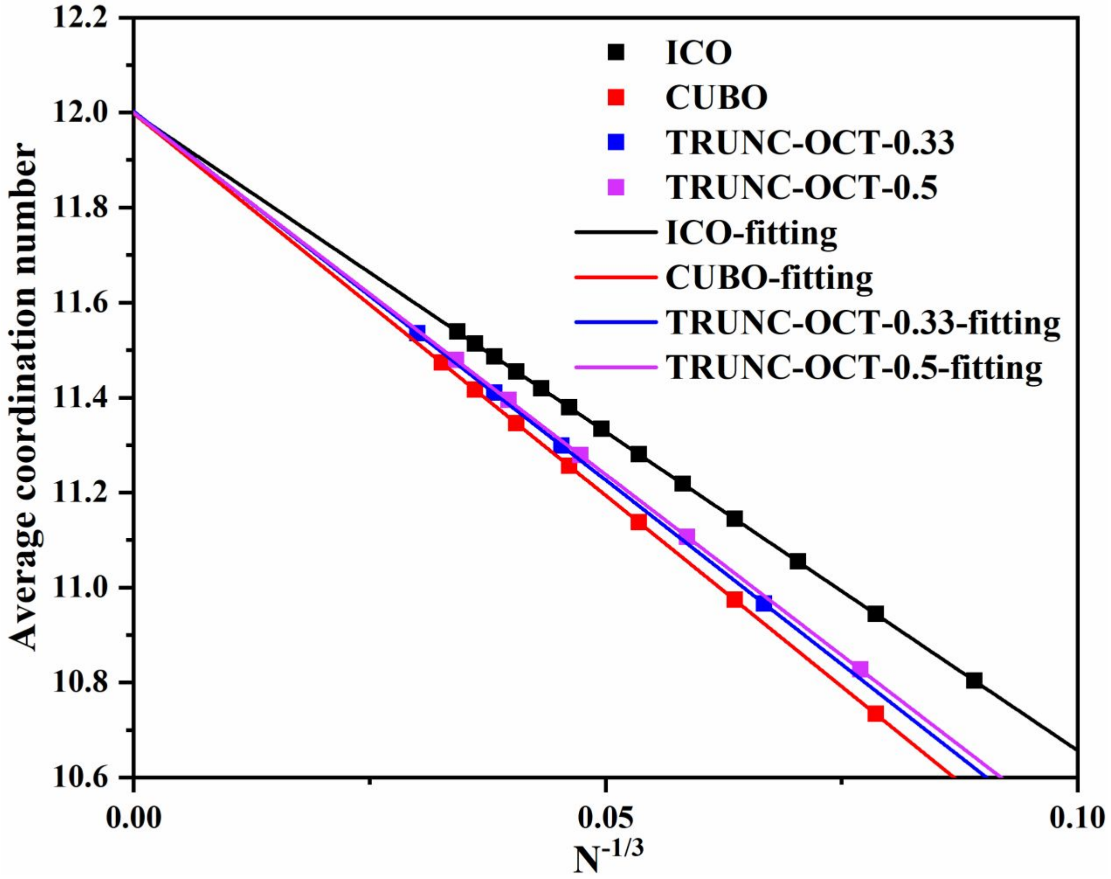

Figure S13. Linear relationship between average coordination number and nanocluster size.
